# The evolutionary consequences of habitat fragmentation: Body morphology and coloration differentiation among brook trout populations of varying size

**DOI:** 10.1002/ece3.3229

**Published:** 2017-07-27

**Authors:** Carol Zastavniouk, Laura K. Weir, Dylan J. Fraser

**Affiliations:** ^1^ Department of Biology Concordia University Montreal QC Canada; ^2^ Department of Biology Saint Mary's University Halifax NS Canada

**Keywords:** genetic drift, natural selection, operational sex ratio, phenotype, salmonid, sexual selection

## Abstract

A reduction in population size due to habitat fragmentation can alter the relative roles of different evolutionary mechanisms in phenotypic trait differentiation. While deterministic (selection) and stochastic (genetic drift) mechanisms are expected to affect trait evolution, genetic drift may be more important than selection in small populations. We examined relationships between mature adult traits and ecological (abiotic and biotic) variables among 14 populations of brook trout. These naturally fragmented populations have shared ancestry but currently exhibit considerable variability in habitat characteristics and population size (49 < *N*_c_ < 10,032; 3 < *N*_b_ < 567). Body size, shape, and coloration differed among populations, with a tendency for more variation among small populations in both trait means and CV when compared to large populations. Phenotypic differences were more frequently and directly linked to habitat variation or operational sex ratio than to population size, suggesting that selection may overcome genetic drift at small population size. Phenotype–environment associations were also stronger in females than males, suggesting that natural selection due to abiotic conditions may act more strongly on females than males. Our results suggest that natural and sexual‐selective pressures on phenotypic traits change during the process of habitat fragmentation, and that these changes are largely contingent upon existing habitat conditions within isolated fragments. Our study provides an improved understanding of the ecological and evolutionary consequences of habitat fragmentation and lends insight into the ability of some small populations to respond to selection and environmental change.

## 1 INTRODUCTION

Human disturbances are resulting in the widespread depletion, fragmentation, and isolation of natural populations (WWF, 2016). As a result, populations can enter an extinction vortex through increased inbreeding and genetic drift, a resulting loss of genetic diversity, and reduced adaptive responses to environmental change (Blomqvist, Pauliny, Larsson, & Flodin, 2010; Gilpin & Soulé, 1986; Hanski & Gilpin, 1991). Yet, before such a genetic extinction vortex commences, emerging evidence suggests that evolution in small populations is highly affected by selective pressures within habitat fragments; these can either improve population persistence or exacerbate extinction risk in the face of future environmental change (Fraser, Debes, Bernatchez, & Hutchings, 2014; Willi, Buskirk, Schmid, & Fischer, 2007; Wood, Belmar‐Lucero, & Hutchings, 2014; Wood, Yates, & Fraser, 2016). Hence, further investigation is required to understand how phenotypic evolution changes among populations as they are fragmented, isolated, and reduced in population size. Such knowledge could allow for differentiating between populations that have or do not have a chance of persisting with resource input.

Both natural selection (defined here as arising from variance in fitness as a result of abiotic factors, in the absence of predators and excluding prey availability) and sexual selection (defined here as arising from variance in mating success) can act on phenotypic traits as a result of specific ecological conditions (Arnold & Wade, 1984; Wellborn & Langerhans, 2015). Strong associations between phenotype and abiotic factors across populations, henceforth phenotype–environment associations, provide support that traits are under natural selection (Langerhans, Chapman, & Dewitt, 2007). Conversely, population differences in secondary sexual characteristics independent of habitat may point to sexual selection (Panhuis, Butlin, Zuk, & Tregenza, 2001). How both natural and sexual selection on phenotypes change when populations have become fragmented, isolated, and small is understudied, but is thought to provide key information on the fate of a population under ongoing environmental change (Franssen, Harris, Clark, Schaefer, & Stewart, 2013; Haugen, Aass, Stenseth, & Vøllestad, 2008; Heinen‐Kay, Noel, Layman, & Langerhans, 2014; Murphy, Battocletti, Tinghitella, Wimp, & Ries, 2016; Palkovacs, Kinnison, Correa, Dalton, & Hendry, 2012).

In stream fishes, abiotic factors such as water temperature, depth, velocity, and pH regularly shape phenotypes. Temperature controls fish metabolism and growth; growth and temperature are positively related (McCormick, Hokanson, & Jones, 1972) as are stream depth and body depth (Quinn, Wetzel, Bishop, Overberg, & Rogers, 2001). Shallower streams are associated with more streamlined, easily maneuverable body shapes in fish, whereas longer pelvic and pectoral fins are expected in deeper streams (Pease, Gonzalez‐Diaz, Rodiles‐Hernandez, & Winemiller, 2012). Higher stream velocity levels are also associated with fusiform body types and longer fins to maintain feeding positions in salmonids specifically (Drinan, McGinnity, Coughlan, Cross, & Harrison, 2012; Riddell & Leggett, 1981). Finally, dark water environments host fish with deeper color to increase visibility to other individuals (Kelley, Bree, Cummins, & Shand, 2012); commonly such waters are low in pH and high in dissolved organic compounds (DOC) in peatland environments (Ishikawa & Gumiri, 2006). Alternatively, decreased water clarity may lead to less coloration as it would not be perceived as readily by other individuals, thus reducing any benefits associated with strong coloration (Ramstad et al. 2010; Seehausen, Van Alphen, & Witte, 1997).

Among stream fish populations, salmonids in particular (salmon, trout, charr) are often physically isolated from one another in different habitats and vary greatly in phenotypic traits and sexual dimorphism (Taylor, 1991; Hutchings, 1996; Riddell & Leggett, 1981; Westley, Conway, & Fleming, 2012). Thus, stream salmonids provide a unique opportunity to understand the natural and sexual selective consequences of habitat fragmentation on phenotypic evolution. Salmonid males compete aggressively for access to females and exhibit exaggerated secondary sexual traits such as a dorsal hump, a deep body shape, and bright coloration in ventral areas, which are indicators of social status, fighting capabilities, and/or intersexual mechanisms for mate attraction (Fleming & Gross 1994; Quinn & Foote, 1994; Blanchfield & Ridgway, 1999; Nitychoruk et al., 2013). In addition, the operational sex ratio (OSR; the ratio of males to females that are ready to mate; Emlen, 1976) is a predictor of the intensity of competition for mates (Emlen & Oring, 1977; Weir, Grant, & Hutchings, 2011) and thus can influence the evolution of secondary sexual characteristics. Collectively, both biotic and abiotic factors are important to consider as putative drivers of selection, as salmonid trait differences often directly relate to individual fitness in local environmental conditions (Fraser, Weir, Bernatchez, Hansen, & Taylor, 2011).

We investigated relationships between adult morphological traits and abiotic and biotic ecological variables among 14 naturally fragmented stream populations of a wild salmonid (brook trout, *Salvelinus fontinalis*). First, we identified whether or not populations differed in body size, shape, and coloration, and examined differences between the sexes in these traits. We then explored possible relationships between phenotype and abiotic habitat factors to determine if population trait differences are putatively driven by natural selection, as well as the influence of OSR on trait differences between the sexes.

**Figure 1:**
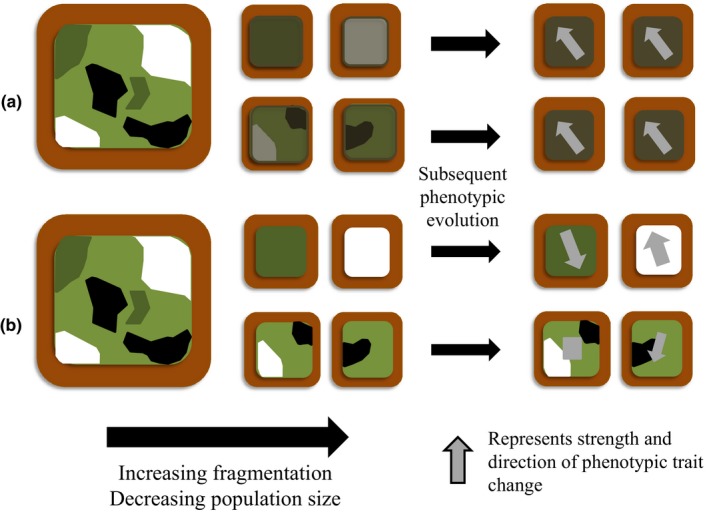
The directional (a) and variable (b) hypotheses. The different shades in the large squares on the left represent habitat types of different qualities and characteristics in an environment. As fragmentation occurs, the directional hypothesis (a) posits that the habitat parameters in each fragment change in a directional way, resulting in similar selection pressures across fragments, for example, through edge effects. When subsequent phenotypic evolution occurs, a directional change occurs in the phenotypic traits across all fragments and populations (modified from Willi & Hoffmann, 2012; Wood et al., 2016). The variable hypothesis (b) posits that habitat quality and characteristics are not changed in a directional way throughout fragmentation and are simply random samples of the habitats found in larger fragments; hence, there are different selection pressures among the fragments. With subsequent phenotypic evolution, each fragment sees a different change in phenotypic trait, both in direction and extent. It is more difficult with the variable hypothesis to systematically predict what will further happen to fragmented populations once they experience large‐scale environmental change (Fraser et al., 2014; Wood et al., 2014, 2016)

We complemented these analyses with two general hypotheses regarding how trait characteristics might change as both habitat fragment size and population size are reduced during habitat fragmentation (see Figure 1). A first, “directional” hypothesis predicts that consistent shifts in habitat characteristics occur during ongoing fragmentation and isolation, and hence so do species traits characteristics (Fraser et al., 2014; Willi & Hoffmann, 2012; Wood et al., 2014, 2016). A second, “variable” hypothesis suggests that habitat characteristics and selection pressures become more variable as fragment and population size decrease, leading to more trait variation among and within small populations (Fraser et al., 2014; Willi et al., 2007; Wood et al., 2014, 2016).

Our study was specifically conducted on isolated brook trout populations in Cape Race, Newfoundland, Canada. These populations were selected because they diverged from a common ancestor during the late Wisconsonian deglaciation (10,000–12,000 ybp; Danzmann, Morgan, Jones, Bernatchez, & Ihssen, 1998) and, much like human‐induced fragmentation, the natural fragmentation experienced by Cape Race populations appears to have arisen rapidly (Fraser et al., 2014) and genetic studies support long‐term isolation (Bernos & Fraser, 2016; Ferguson, Danzmann, & Hutchings, 1991; Fraser et al., 2014; Wood et al., 2014). Previous research has also suggested that standing neutral genetic diversity within these populations is directly proportional to population size (Wood et al., 2014). These populations vary 200‐fold in census population size (*N*_c_) and 100‐fold in their effective number of breeders (*N*_b_—analogous to the effective population size *N*_e_ but for an individual cohort) (Table S3; Bernos & Fraser, 2016). Population sizes reflect those that are typically very small to very large for vertebrates, including several below minimum viable population sizes for conservation (Reed, O'Grady, Brook, Ballou, & Frankham, 2003); thus, our study's results may have general implications for fragmented and isolated vertebrate populations.

## 2 MATERIALS AND METHODS

### 2.1 Study site

Cape Race is a barren coastal region situated in south‐eastern Newfoundland, Canada (Fig. S1). Multiple brook trout populations persist within a parallel series of relatively short, low‐order streams (0.27–8.10 km), which create an ideal environment for investigating phenotypic and breeding traits in fragmented vertebrate populations of varying size (49 < *N*_c_ < 10,032; 3 < *N*_b_ < 567; Bernos & Fraser, 2016). Due to their small size, Cape Race streams can be sampled comprehensively to obtain reliable population size estimates. The trout populations are pristine in having no history of stocking, and little to no angling pressure due to the small body size of the fish. The populations are all genetically distinct; most completely isolated by inhabiting streams that terminate as 30–50 m waterfalls directly into the sea. Exceptions are the pairs BF‐WN, DY‐UO, and DY‐UO for which occasional gene flow occurs (Wood et al., 2014; Wood, Tezel, Joyal, & Fraser, 2015; Fig. S1).

### 2.2 Data collection

From September through October 2014, we photographed 1,059 fish for phenotypic analyses described below from 14 Cape Race populations with multiyear population size and habitat data (Bernos & Fraser, 2016). Individuals were randomly sampled throughout streams using a backpack electrofisher; however, spawning aggregates were targeted in those streams where they were found. Only fish that were reproductive that season were chosen for photographs; immature individuals and postspawn females were not included. This selection was performed by assessing individuals visually and physically for determining spawning readiness (i.e., gently squeezing abdomens). Each population was sampled during its spawning period (at a similar ratio of ready females to not ready females), allowing for standardized interpopulation comparisons.

#### 2.2.1 Morphological traits

##### Body size and shape

We compared body size and shape between populations using length and mass measurements and photography. Five fish were anesthetized at a time using tricaine mesylate (MS‐222) at 0.2 g/L. A wooden platform was used to ensure a level tripod and camera, which was set up at the same angles, distance, and zoom for each picture. A size reference, ruler, and individual label were included and placed in a similar position in each photograph. The sex, spawning readiness, length, and mass were collected. Fish were then placed in a recovery container for 10 min before release back into the stream. Condition factor was calculated using the formula $K\;=\;\frac{10^{5}\;\times \;\text{Mass}}{{\text{Length}}^{3}}$ (Weatherly & Gill, 1987).

To calculate body shape, geometric morphometric analysis was conducted. In each photograph, seventeen landmarks were placed along the fish outline and assigned an x,y coordinate to produce a consensus shape using the program tpsDig2 (v.154, Rohlf, 2014; see Fig. S2). These landmarks were then used to produce relative warps (RWs), a multivariate description of shape variation, through tpsRelw (v.154) (Bookstein, 1991; Rohlf, 2014; Zelditch, Swiderski, Sheets, & Fink, 2004). The first four of thirty total RWs were used for statistical analysis of body shape, as these explained most (64.6%) of the total variation and were related to secondary sexual characteristics. ImageJ (Schneider, Rasband, & Eliceiri, 2012) was used to measure pelvic and pectoral fin length, measured as the maximum distance from the proximal to distal margin of the fin (Pease et al., 2012).

##### Body color

Redness in body color (total area and saturation) was compared between populations. Inclusion of a X‐Rite^®^ ColorChecker Passport (a color palette used as a standardization tool) in each photograph allowed for the removal of any changing lighting conditions, using nip2 (VIPS software; Martinez & Cupitt, 2005). Following the methods of Wedekind, Jacob, Evanno, Nusslé, & Müller, 2008 (using ImageJ), the total area of redness and its saturation level was calculated on each individual (excluding dorsal, adipose, and anal fins). We also used ImageJ to count red spots with blue halos on fish abdomens (limited to below the lateral line to reduce confounding from glare).

#### 2.2.2 Abiotic habitat characteristics

Summer habitat variables were taken from stream measurements during mid‐June to mid‐July in 2012–2015, from 19 to 64 transects per stream (uniformly along the entire length of fish sampling areas). We considered the following variables: water temperature, pH, velocity, and depth. A WTW Multiline P4 universal meter was used to measure temperature and pH. Velocity (m/s) was measured by releasing a ball attached to a one meter string from an upstream position and recording the time required for the ball to travel one meter with the current. Mean velocity and depth per transect (measured using a meter stick to a precision of 0.1 mm) were calculated as the average of three to six measurements spaced equally across the width of the stream channel. Overall habitat means within streams were calculated by bootstrapping values to account for differences in sampling effort between years (ensuring that all years were weighted equally).

#### 2.2.3 Biotic habitat characteristics

2.2.3.1

###### Operational sex ratio

We calculated operational sex ratio (OSR) as the ratio of potentially receptive males to potentially receptive females in a population (fish that were classified as “almost ready” and “ready”) (sensu Emlen, 1976). Potentially receptive individuals were determined from stream surveys assigning spawning readiness for each fish caught (i.e., not close/almost ready/ready/spawned for females, and ready/not ready for males, with “not ready” meaning several days to weeks away from spawning and “almost ready” meaning spawning would happen within 1–4 days). Average OSR was used as datum for each population.

###### Population size

Mean population estimates for *N*_c_ and *N*_b_ were taken from Bernos and Fraser (2016). Harmonic means were used to ensure averages that were not biased by outliers. Additional calculations were carried out to obtain an *N*_b_ estimate for two populations (FW and PD), using a model describing the relationship between *N*_c_ and *N*_b_ in Cape Race trout populations (see Bernos & Fraser, 2016, Table 2, Full N model).

### 2.3 Trait analysis

#### 2.3.1 Interpopulation trait variation

We used linear models to compare trait differences between populations (in R Studio 0.99.484, R Core Team, 2015). Body size, shape, and color data were firstly tested for normality within each population using a Shapiro–Wilks test and by examining residual distributions. Mass and condition factor were log transformed as they were non‐normal in several populations. Red area (area of red color/total body area) was analyzed using a beta regression to account for data over‐dispersion and heteroscedasticity (Cribari‐Neto & Zeileis, 2009). Independent predictor variables in our linear models were population, sex, and a population × sex interaction, tested through backwards stepwise model selection. Centroid size, a geomorphometric measure of overall body size, was included in our models as a covariate, but was removed from body size models to avoid redundancy due to a high correlation with mass and length. For traits with a significant population × sex interaction, least‐square means (R package lsmeans; Lenth, 2016) were used to identify significant differences between population, sex, as well as within‐population sex differences. Statistical significance levels were adjusted to control for type I error via a FDR correction, and also divided into half (*p* < .025) for length and mass to account for their nonindependence.

#### 2.3.2 Phenotype–environment associations

We tested whether mean stream habitat variables were putative drivers of interpopulation variation in body size, shape, and color using linear mixed models (LMMs). Habitat variables were centered around zero. Collinearity between variables was tested through variance inflation factors (vif); those variables with vif values higher than 5 were discarded (two interactions: stream depth × velocity and depth × temperature). Interactions between habitat variables that were not collinear and biologically relevant were included in LMMs, specifically stream pH × temperature, and stream temperature × velocity. Population size (to test the directional hypothesis, *N*_c_ and *N*_b_ in separate analyses), sex (to account for putative differences between sexes), and a random effect of population (to control for population level variation) were also included in the models. Backwards stepwise model selection was conducted for each trait individually. As red area/total body area is proportional data, a logit transformation was performed prior to modeling to create continuous values along a real line [‐inf, inf] instead of proportions [0,1]. For those models that showed a significant difference in sex, within‐sex models were used to test for population differences within each sex separately.

The possibility of using a multivariate analysis instead of a univariate analysis (as described above) was explored but ultimately rejected due to issues with interpretation. Specifically, we conducted a Principal Components Analysis (PCA) on the 12 variables of interest. When replacing dependent variables with Principal Components (PCs), 80% of the variance should be explained (Crawley, 2007); in our data, seven PCs were needed to explain 80% of the variance for the 12 phenotypic traits. The patterns of relationships among the PCs and the different trait variables were not amenable to ease of interpretation; while some pairs of trait variables showed similar patterns, most only highly influenced one of the possible PCs, suggesting that this approach did not lend itself to increasing the ease of reporting our data.

#### 2.3.3 Directional and variable hypotheses

The directional hypothesis was first tested for each trait using linear models described above. We also visually assessed plots of phenotypic traits against *N*_b_ and *N*_c_ to find corresponding patterns relating to consistent (directional hypothesis) or more variable (variable hypothesis) trait changes with population size, using both trait means and coefficients of variation (CV; a normalized measure of dispersion where CV = σ/μ [standard deviation/mean]). To further test whether trait variability (both trait means and CVs) specifically increased at smaller population sizes, we used White's test for heteroscedasticity (White, 1980).

## 3 RESULTS

### 3.1 Inter‐population and inter‐sex trait variation

**Figure 2:**
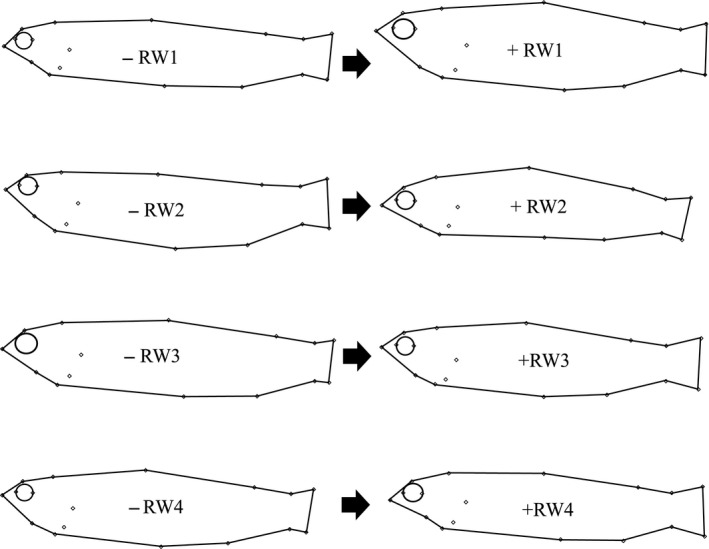
Extreme positive and negative shapes for RW1‐4, across 14 brook trout populations from Cape Race, Newfoundland, Canada. Variance explanation from each warp is as follows: RW1 29.32%, RW2 16.32%, RW3 10.93%, RW4 8.03%. From negatives values on the left to positive on the right: RW1 shows increase in body depth, RW2 shows horizontal alignment change going from extended ventral side to extended dorsal side, RW3 shows caudal peduncle increasing compared to torso length, and RW4 shows mouth angle increase, decrease body depth, and head narrowing

Details of percent variation explained at each RW and average consensus shapes are found in Figure 2. RW1 explains a gradient in body depth, head size, and eye size, RW2 shows horizontal alignment change (extended ventral side or dorsal side), RW3 explains caudal peduncle shape and length compared to torso length, and RW4 explains head shape and snout angle.

All twelve morphological traits assessed were significantly different among populations (*p* < 2.20E‐16; Table 1), ranging from a 1.32‐fold mean difference in red saturation (106.73 units to 141.49 units, Table S1) to a 2.96‐fold mean difference in mass (13.35–39.51 g; Table S1). Across populations, 10 of 12 traits also varied significantly between sexes (*p* < .001) (exceptions were the number of red spots and pelvic fin length; Table 1), ranging from a 1% mean difference in condition factor (female 1.179 to male 1.184) to a 15% mean difference in red saturation (female 118.89 units to male 138.30 units; Table S2). In most cases, males had greater trait values than females (exceptions: mass, length, and RW4). *F*‐values were much higher for population than sex for 9 of 12 traits, indicating that among‐population differences were much larger than sex differences (exceptions are RW1, RW2, and red color saturation; Table 1).

**Table 1:** *F*‐values (***p < .001, **p < .01, *p < .05, NS *p* > .05) of all traits in relation to each tested variable, using linear models (or a beta regression model for red area)

Trait Category	Trait	Population (df = 13)	Sex (df = 1)	Centroid Size (df = 1)	Pop:Sex (df = 13)
Body size	Mass	*F* = 64.491***	*F* = 8.939**	N/A	NS
Body size	Length	*F* = 64.743***	*F* = 11.616**	N/A	NS
Body size	Condition factor	*F* = 14.768***	*F* = 10.981**	N/A	*F* = 2.078*
Body shape	RW1	*F* = 14.768***	*F* = 1005.243***	*F* = 52.690***	*F* = 6.417***
Body shape	RW2	*F* = 30.285***	*F* = 64.836***	*F* = 29.011***	NS
Body shape	RW3	*F* = 55.660***	*F* = 59.529***	*F* = 16.942***	NS
Body shape	RW4	*F* = 65.044***	*F* = 5.296*	*F* = 65.533***	*F* = 1.958*
Coloration	Red Area	*F* = 50.899***	*F* = 15.335***	*F* = 50.646***	*F* = 3.284***
Coloration	Red Saturation	*F* = 34.839***	*F* = 382.549***	*F* = 4.795*	*F* = 5.330***
Coloration	Spot number	*F* = 64.772***	NS	*F* = 211.691***	NS
Fin Length	Pectoral Fin	*F* = 390.201***	*F* = 69.185***	*F* = 3501.326***	*F* = 3.087**
Fin Length	Pelvic Fin	*F* = 240.622***	NS	*F* = 2379.589***	*F* = 1.993*

**Figure 3:**
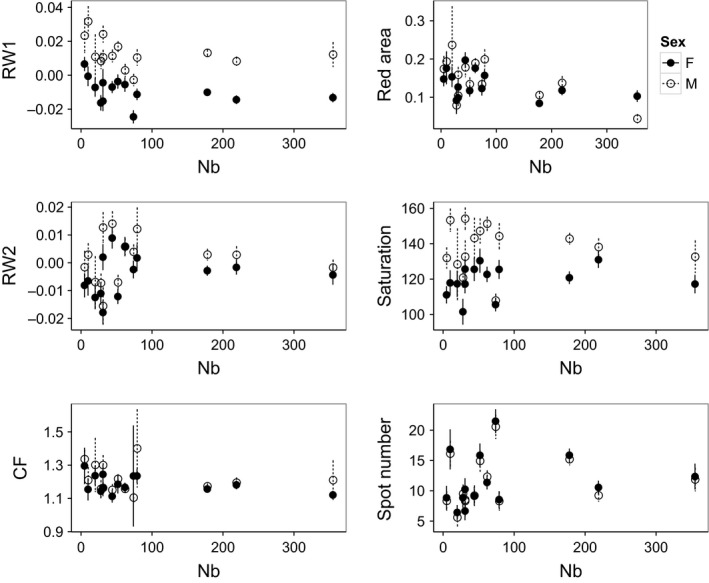
Female and male means of traits that support the variable hypothesis (more variability in small populations), from left to right: RW1 (body depth), red area/total body area, RW2 (dorsal hump), red saturation, condition factor (CF), and spot number across 14 brook trout populations in Cape Race, Newfoundland, Canada, increasing in population size (*N*_b_) along the x‐axes. Fig. S3 shows remaining traits. Trait means depicted with 95% confidence intervals

*F*‐values for the population × sex interaction were much lower than those for population and sex separately, but this interaction was significant for 7 of 12 traits (Table 1). Of these traits, three or more populations had sex differences inconsistent with the general trend, driving the interaction (see Figures 3 and S3 for examples); for body depth (RW1), only one population's males were not much deeper than females to primarily drive the interaction (Figure 3).

### 3.2 Phenotype–environment associations

**Figure 4:**
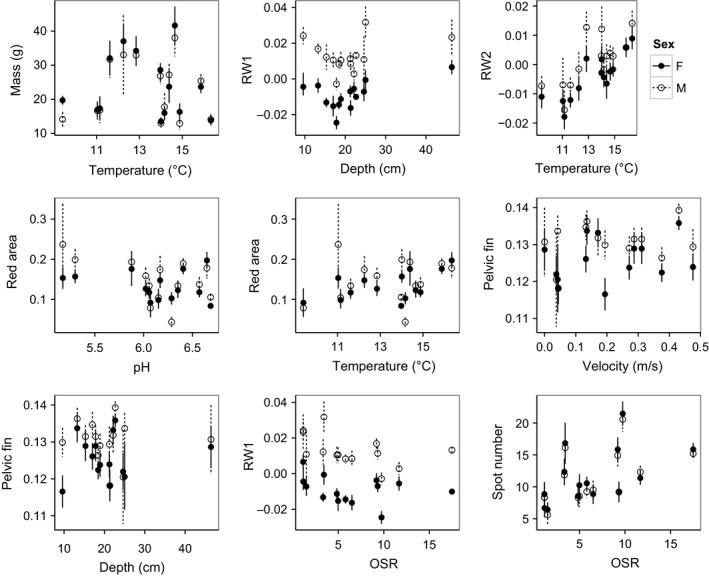
Examples of mean trait and abiotic habitat interactions in 14 brook trout populations in Cape Race, Newfoundland, Canada. From left to right: mass across stream temperatures, RW1 (body depth) across stream depth, RW2 (dorsal hump) across stream temperatures, red area/total body area across stream pH, red area/total body area across stream temperatures, pelvic fin length/total body length across stream velocities, pelvic fin length/total body length across stream velocities, RW1 (body depth) across stream OSRs, and spot number across steam OSRs. Trait means depicted with 95% confidence intervals

Biologically interpretable phenotype–environment associations were detected in all 12 traits; out of a possible 224 phenotypic trait vs. habitat or ecological variable comparisons, 61–73 were significant (*p* < .05) (Tables 2, S4, S5). Plots of significant phenotype–environment associations can be found in Figure 4; additional associations can be found in Fig. S4. Trout were significantly larger in warmer and slower streams, although these relationships appear to be weak. A larger dorsal hump/small ventral extension (RW2) was strongly associated with warmer water. Body redness was greater in acidic streams for both sexes, and female redness increased in deeper, faster, and warmer streams. Fast streams also favored longer pectoral and pelvic fin lengths in females more strongly, and only pelvic fin length was positively associated with depth in females. Body size had a weak positive relationship with average OSR in females, body depth (RW1) for males decreased with increasing OSR, and head size (RW4) decreased with increasing OSR. Pelvic fin length was strongly positively associated with higher OSR in females, and number of spots also increased with OSR.

**Table 2:** Linear mixed models of best fit for each phenotypic trait, with habitat characteristics, sex, number of breeders (*N*_b_), and OSR as predictor variables in 14 brook trout populations in Cape Race, Newfoundland, Canada. An appropriate measurement of body size was added as a correlate where applicable. Condition factor and spot number have overall results only as sex was not significant. Models were performed for both sexes combined (indicated with “O”) as well as separated (indicated with “F” and “M”) and tested using likelihood ratio tests

Phenotypic trait	Model of best fit for phenotype–environment associations
Mass	
O	lmer(log(Mass) ~ Temperature + Velocity + Temperature:Velocity + Sex + (1|Population))
F	lmer(log(Mass) ~ Temperature + Velocity + Temperature:Velocity + OSR + (1|Population))
M	lmer(log(Mass) ~ Temperature + Velocity + Temperature:Velocity + (1|Population))
Length	
O	lmer(Length ~ Temperature + Velocity + Temperature:Velocity + Sex + OSR + (1|Population))
F	lmer(Length ~ Temperature + Velocity + Temperature:Velocity + OSR + (1|Population))
M	lmer(Length ~ Temperature + Velocity + Temperature:Velocity + (1|Population))
Condition factor	
O	lmer(log(Condition Factor) ~ pH + (1|Population))
RW1	
O	lmer(RW1 ~ log(Centroid.size) + Sex + OSR + (1|Population))
F	lmer(RW1 ~ log(Centroid.size) + (1|Population))
M	lmer(RW1 ~ log(Centroid.size) + OSR + (1|Population))
RW2	
O	lmer(RW2 ~ log(Centroid.size) + Temperature + Sex + (1|Population))
F	lmer(RW2 ~ log(Centroid.size) + Temperature + (1|Population))
M	lmer(RW2 ~ log(Centroid.size) + Temperature + (1|Population))
RW3	
O	lmer(RW3 ~ log(Centroid.size) + Sex + (1|Population))
F	lmer(RW3 ~ log(Centroid.size) + (1|Population))
M	lmer(RW3 ~ log(Centroid.size) + (1|Population))
RW4	
O	lmer(RW4 ~ log(Centroid.size) + Depth + Sex + Nb + OSR + (1|Population))
F	lmer(RW4 ~ log(Centroid.size) + Nb + OSR + (1|Population))
M	lmer(RW4 ~ log(Centroid.size) + Depth + Nb + OSR + (1|Population))
Red area	
O	lmer(logit(Red area) ~ log(Body.area) + Depth + Temperature + Sex + Nb + (1|Population))
F	lmer(logit(Red area) ~ log(Body.area) + pH:Temperature + Temperature:Velocity + pH + Depth + Velocity + Temperature + Nb + (1|Population))
M	lmer(logit(Red area) ~ log(Body.area) + pH + Temperature + Sex + Nb + (1|Population))
Red saturation	
O	lmer(Saturation ~ log(Body.area) + Sex + (1|Population))
F	lmer(Saturation ~ log(Body.area) + (1|Population))
M	lmer(Saturation ~ log(Body.area) + Nb + (1|Population))
Spot Number	
O	lmer(Spot number ~ OSR + (1|Population))
Pectoral Fin	
O	lmer(Pectoral fin ~ log(Body.length) + Sex + (1|Population))
F	lmer(Pectoral fin ~ log(Body.length) + Temperture:Velocity + Depth + Velocity + Temperature + Sex + (1|Population))
M	lmer(Pectoral fin~ log(Body.length) + (1|Population))
Pelvic Fin	
O	lmer(Pelvic fin~ log(Body.length) + Temperature:Velocity + Temperature + Velocity + Sex + (1|Population))
F	lmer(Pelvic fin~ log(Body.length) + Temperature:Velocity + Depth + Temperature + Velocity + Nb + (1|Population))
M	lmer(Pelvic fin~ log(Body.length) + Temperature:Velocity + Temperature + Velocity + (1|Population))

Overall, females had more significant differences (24 vs. 16) and generally stronger relationships between phenotypic traits and habitat variables than males, suggesting stronger phenotype–environment associations. Of the significant trait‐habitat combinations in both sexes, *F*‐values were higher in females in 8 of 13 cases (Tables 2, S4).

### 3.3 Directional and variable hypotheses

**Figure 5:**
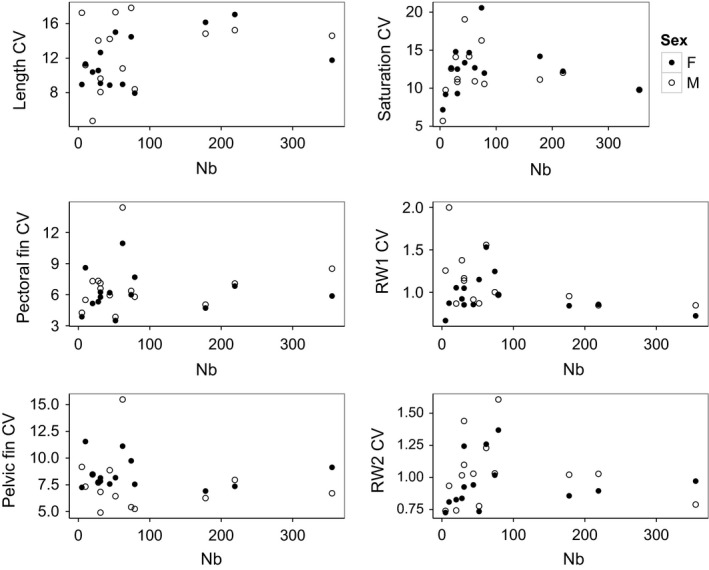
Coefficient of variation (CV) of traits by sex against population size (*N*_b_) in 14 brook trout populations in Cape Race, Newfoundland, Canada. From left to right: length, pelvic fin length/total body length, red saturation, RW1 (body depth), pectoral fin length/total body length, RW2 (dorsal hump). Of twelve traits, these six showed semblance to the variable hypothesis (more variability in small populations)

Only two traits (red area and RW4) showed a pattern consistent with the directional hypothesis (a positive or negative association was observed between each trait and *N*_c_ or *N*_b_; Tables 2, S4, S5). Conversely, 6 of 12 traits showed the pattern expected through the variable hypothesis for both trait means and trait CV when plotted against *N*_b_ (trait means: red area/total body area, red saturation, RW1, RW2, condition factor, spot number; trait CV: red saturation, RW1, RW2, length, pectoral fin size, pelvic fin size; Figures 3 and 5, respectively). However, White's tests did not show statistically significant heteroscedasticity among population size (*N*_c_ or *N*_b_) for any trait (means or CVs) (Appendix S3).

## 4 DISCUSSION

We found a large number of phenotype–environment associations in fragmented brook trout populations, consistent with the hypothesis that selection may be influencing interpopulation differences in adult morphological traits. We also found little support that population size (and by extension the amount of genetic variation) affects sexual trait characteristics in these isolated populations. Previous studies (Fraser et al., 2014; Wood et al., 2014, 2015) found that across Cape Race trout populations (including the 14 in this study), the process of habitat fragmentation increased variability in spatial habitat, adaptive genetic differentiation, and in early life traits (morphology, behavior, growth) going from large to small fragment and population size, consistent with the variable hypothesis. Our results suggest that although there appears to be some semblance of more variation in the adult size, shape, and coloration traits in small populations, existing habitat characteristics can better explain trends in adult characteristics among Cape Race trout populations than the variable or directional hypotheses. This may be due to genetic and/or environmental factors that operate differentially at different life stages in brook trout. Additionally, with only 14 populations, we might have had reduced statistical power for detecting significant trait heteroscedasticity in relation to population size. To some extent, the contrast between life stages may also be due to phenotypic plasticity within each population in response to habitat variables (e.g., head and eye size are known to be plastic with growth rate; Devlin, Vandersteen, Uh, & Stevens, 2012). Nevertheless, the associations between phenotypes and environments, combined with the large number of genetically based trait differences observed among Cape Race populations using common garden experimentation, suggest that body size, shape, and color traits are under selection (Wood & Fraser, 2015; Wood et al., 2015; Wells, McDonnell, Chapman, & Fraser, 2016; D. Fraser, unpublished results, see Appendix S4 in Supplementary Materials for a population comparison between wild and captive body size).

Our results have three key implications. First, they suggest that natural selection on adult traits in fragmented populations may operate even under conditions of pronounced genetic drift (mean *N*_b_ ranging from 5 to 355, mean *N*_e_ 9 to 589 with four populations *N*_e_ < 50 and five populations 50 < *N*_e_ > 100; Bernos & Fraser, 2016). This is consistent with recent meta‐analysis findings across taxa (Wood et al., 2016) and further supports Fisherian evolutionary theory (purporting that phenotypic differentiation primarily results from positive natural selection; Fisher, 1930; see also Koskinen, Haugen, & Primmer, 2002). Second, the strength and number of phenotype–environment associations were higher in females than in males, suggesting that female phenotypic traits may be under a stronger influence from natural selection. Lastly, across a fragmented landscape of many populations, trait differentiation that appears to be influenced by natural selection—a deterministic process—also appears to be highly influenced by starting conditions of initial fragmentation events that are largely random with respect to habitat patch characteristics and population size (see also Figure 1).

As expected based on previous work (Belmar‐Lucero et al., 2012; Hutchings, 1993, 1996)**,** Cape Race trout populations were highly differentiated for all 12 traits despite occupying a very small geographic scale. Phenotype–environment associations observed were consistent with theoretical expectations and previous works on stream fishes (Ishikawa & Gumiri, 2006; Kelley et al., 2012; McCormick et al., 1972; Pease et al., 2012; Riddell & Leggett, 1981), again suggesting that traits are under selection in all Cape Race populations despite their large spread in population size. However, many predicted associations were seen in females only. For example, deeper streams yielded female fish with redder bodies and longer fins, and warmer streams yielded larger fish overall (although this relationship was not strong, perhaps because warmer waters are associated with greater growth rate and not overall size). We also found higher amounts of red body coloration for both sexes in populations inhabiting acidic streams, corresponding to theoretically increased DOC levels (Ishikawa & Gumiri, 2006). This is in agreement with Kelley et al. (2012) who found that fish with more intense coloration are associated with darker water. Females, more than males, showed the predicted relationship with pelvic and pectoral fin length: Fish in deeper streams had longer pelvic fins and both pelvic and pectoral fins were longer in faster streams. However, a relationship between body depth and stream depth was not found, although it appears there is a positive association after 18 cm or more of stream depth. This lack of relationship at shallower depths is most likely caused by the stream LC, which has the shallowest average depth but also has several deep pools where fish reside (Figure 4, top panel centre; Table S3). Additionally, a strong relationship was found between warm water and body redness. This corresponds to previous studies finding higher carotenoid concentrations in goldfish inhabiting warmer water (Gouveia & Rema, 2005) and a higher apparent digestibility coefficient of astaxantin (a carotenoid) in Atlantic salmon reared at 12°C versus those reared at 8°C (Ytrestøyl, Struksnæs, Koppe, & Bjerkeng, 2005).

Although salmonids generally exhibit elaborate sexual dimorphism (Nitychoruk et al., 2013; Weir, Kindsvater, Young, & Reynolds, 2016; Young, 2005), population explained more variation than sex in Cape Race trout (Table 1). Natural and sexual selection occur concurrently in many vertebrates (Crothers & Cummings, 2013; Johnson, 2001; Langerhans & Dewitt, 2004; Romano, Costanzo, Rubolini, Saino, & Møller, 2016) and can also operate with different intensities between sexes to shape breeding behavior and tactics (Dunn, Armenta, & Whittingham, 2015; Fleming, 1998). Results from our study are consistent with these findings by showing that phenotypic traits in females are in general more a function of habitat characteristics than those of males. This may mean that females are under greater natural selection while mating competition continues to drive sexual selection in males. Both parallel and nonparallel evolution of the sexes has been shown to occur in vertebrates in response to habitat variables (Berns & Adams, 2013; Butler, Sawyer, & Losos, 2007; Hendry, Kinnison, & Reznick, 2006). In Cape Race brook trout, males and females exhibited similar trends in the direction that habitat variables acted upon traits, but the strength of phenotype–environment associations was greater in females.

Sexual selection may also differ in populations as a function of the environment, with ecological variation altering the context of sexual selection (Anderson & Langerhans, 2015; Romano et al., 2016). This can be seen with body depth in male sockeye salmon; deep‐bodied males in deep‐water environments are dominant, while the dominant males in shallow water are not significantly deeper‐bodied (Hamon & Foote, 2005). Of the 12 traits, those showing high sexual dimorphism (higher values for body depth, dorsal hump, and red color saturation among males) are typical of secondary sexual characteristics found in salmonids and are important indicators of sexual selection (Quinn & Foote, 1994). However, each of these traits was still influenced by habitat and differed among populations. Although RW2 can be an indication of an arch effect (slight posture differences between individuals during photography; Valentin, Penin, Chanut, Sévigny, & Rohlf, 2008), males in our study consistently had higher values, or higher dorsal arch, than females. This supports that RW2 in our study was, at least in part, an indication of a sexual trait. Lower values also represented extended ventral areas which would be typical of mature females carrying eggs during their spawning season.

Traits known to be sexually selected in salmonids (e.g., body size and depth) were also influenced by the OSR. Male competition is highest at a male‐biased OSR of 1–4, at which sexually selected traits should be the most exaggerated. A higher OSR could potentially decrease the rate of competition as the ratio of effort to outcome becomes more skewed (Quinn, Adkison, & Ward, 1996), including sperm competition (Pilastro, Scaggiante, & Rasotto, 2002). This can be seen with male (and to a lesser extent, female) body depth (RW1) in Cape Race populations; as the ratio of males to females increases (OSR ranging from 1.15 to 9.90), body depth decreases (Table S4). As for other secondary sexual characteristics, this relationship was also not seen with body size or redness in both sexes, perhaps as a result of stronger selection from habitat variables.

Although small and large populations did not consistently differ across most trait characteristics, two traits (red area over total body area and RW4) were shown to significantly change with population size, although the relationship with RW4 was not strong (Table S4). In the case of red coloration, this negative relationship may be a function of increased sexual selection in smaller populations, as competition remains high because of smaller or no spawning aggregates (OSR 1–4) (Quinn et al., 1996). Females may be more selective in choosing a more colorful male in those populations where there are no aggregates, as four of the five largest populations have a very high OSR (Table S3) and three of five have large aggregations during peak spawning period (personal observations). We hypothesize that the directional relationship seen between population size and red coloration is therefore a function of sexual selection, in lieu of accrued genetic drift.

### 4.1 Evolutionary ecology and conservation implications

The full genetic and evolutionary consequences of landscape modification remain understudied (Fischer & Lindenmayer, 2007). Although habitat fragmentation and subsequent population size reduction can reduce genetic diversity within populations (Alò & Turner, 2005; Blanchet, Rey, Etienne, Lek, & Loot, 2010), these changes can also alter selective pressures, with subsequent effects on population persistence, before classic extinction vortexes might ensue (Fraser et al., 2014; Wood et al., 2014). We attempted to address this research gap by comparing 14 naturally fragmented trout populations and found that population size and genetic variation are less important indicators of morphological variability in body size, shape, and color in both sexes, compared to existing habitat characteristics within fragments. This suggests that Cape Race populations exhibit environmentally selected characteristics despite a potential lack of genetic diversity due to small population size. Nevertheless, while selection appears to overcome drift in large to small Cape Race populations, it also appears to be highly contingent upon random starting conditions in each habitat fragment. Some populations appear to become fragmented in marginal, poor quality habitats while others become isolated in fragments of high quality but that simply have a small finite size—and changing habitat characteristics with ongoing fragmentation can favor the maintenance of genetic diversity in some small populations rather than reducing it (Fraser et al., 2014; Wood et al., 2014, 2016).

Through similar studies to this one, it is becoming apparent that fine‐scale local adaptation might play an important part in maintaining small, isolated populations. Forecasting traits and genetic makeup based on population size may not adequately predict the variation that is observed (Giery, Layman, & Langerhans, 2015; Letcher, Nislow, Coombs, O'Donnell, & Debreuil, 2007; Wood et al., 2016). As anthropogenic disturbances escalate in scale and rate causing decreases in habitat and population sizes, it may therefore be difficult to predict trajectories of populations at a large geographic scale. Case‐by‐case consideration of each habitat fragment and the population inhabiting it may be critical. In the face of resource‐limited conservation, a potentially effective method might be to prioritize those populations that (i) have experienced very small population sizes yet maintain relatively high genetic and phenotypic variation and (ii) are experiencing more similar environmental conditions to those presumably faced in the future with climate change.

## CONFLICT OF INTEREST

None declared.

## CONTRIBUTIONS OF AUTHORS

C. Zastavniouk conducted fieldwork, statistical analyses, and redaction of the manuscript. D.J. Fraser provided field and logistical support, theoretical guidance, and writing assistance. L. K. Weir provided theoretical guidance and writing assistance.

## References

[ece33229-bib-0001] Alò, D. , & Turner, T. F. (2005). Effects of habitat fragmentation on effective population size in the endangered Rio Grande silvery minnow. Conservation Biology, 19(4), 1138–1148.

[ece33229-bib-0002] Anderson, C. M. , & Langerhans, R. B. (2015). Origins of female genital diversity: Predation risk and lock‐and‐key explain rapid divergence during an adaptive radiation. Evolution, 69(9), 2452–2467. 26259062 10.1111/evo.12748

[ece33229-bib-0003] Arnold, S. J. , & Wade, M. J. (1984). On the measurement of natural and sexual selection: Applications. Evolution, 38(4), 720–734. 28555830 10.1111/j.1558-5646.1984.tb00345.x

[ece33229-bib-0004] Belmar‐Lucero, S. , Wood, J. L. A. , Scott, S. , Harbicht, A. B. , Hutchings, J. A. , & Fraser, D. J. (2012). Concurrent habitat and life history influences on effective/census population size ratios in stream‐dwelling trout. Ecology and Evolution, 2, 562–573. 22822435 10.1002/ece3.196 PMC3399145

[ece33229-bib-0005] Bernos, T. A. , & Fraser, D. J. (2016). Spatiotemporal relationship between adult census size and genetic population size across a wide population size gradient. Molecular Ecology, 25, 4472–4487. 27483203 10.1111/mec.13790

[ece33229-bib-0006] Berns, C. M. , & Adams, D. C. (2013). Becoming different but staying alike: Patterns of sexual size and shape dimorphism in bills of hummingbirds. Evolutionary Biology, 40(2), 246–260.

[ece33229-bib-0007] Blanchet, S. , Rey, O. , Etienne, R. , Lek, S. , & Loot, G. (2010). Species‐specific responses to landscape fragmentation: Implications for management strategies. Evolutionary Applications, 3(3), 291–304. 25567925 10.1111/j.1752-4571.2009.00110.x PMC3352461

[ece33229-bib-0008] Blanchfield, P. , & Ridgway, M. (1999). The cost of peripheral males in a brook trout mating system. Animal Behaviour, 57(3), 537–544. 10196043 10.1006/anbe.1998.1014

[ece33229-bib-0009] Blomqvist, D. , Pauliny, A. , Larsson, M. , & Flodin, L. (2010). Trapped in the extinction vortex? Strong genetic effects in a declining vertebrate population. BMC Evolutionary Biology, 10, 33. 20122269 10.1186/1471-2148-10-33 PMC2824661

[ece33229-bib-0010] Bookstein, F. L. (1991). Morphometric tools for landmark data: Geometry and biology. New York, NY: Cambridge University Press.

[ece33229-bib-0011] Butler, M. A. , Sawyer, S. A. , & Losos, J. B. (2007). Sexual dimorphism and adaptive radiation in Anolis lizards. Nature, 447(7141), 202–205. 17495925 10.1038/nature05774

[ece33229-bib-0012] Crawley, M. J. (2007). Multivariate statistics, in the R book. Chichester, UK: John Wiley & Sons Ltd https://doi.org/10.1002/9780470515075.ch23

[ece33229-bib-0013] Cribari‐Neto, F. , & Zeileis, A. (2009). Beta regression in R. *Research Report Series/Department of Statistics and Mathematics*, 98 (pp.1–22).

[ece33229-bib-0014] Crothers, L. , & Cummings, M. (2013). Warning signal brightness variation: Sexual selection may work under the radar of natural selection in populations of a polytypic poison frog. The American Naturalist, 181, 116–124. 10.1086/670010 23594556

[ece33229-bib-0015] Danzmann, R. G. , Morgan, R. P. , Jones, M. W. , Bernatchez, L. , & Ihssen, P. E. (1998). A major sextet of mitochondrial DNA phylogenetic assemblages extant in eastern North American brook trout (*Salvelinus fontinalis*): Distribution and postglacial dispersal patterns. Canadian Journal of Zoology, 76, 1300–1318.

[ece33229-bib-0016] Devlin, R. H. , Vandersteen, W. E. , Uh, M. , & Stevens, E. D. (2012). Genetically modified growth affects allometry of eye and brain in salmonids. Canadian Journal of Zoology, 90(2), 193–202.

[ece33229-bib-0017] Drinan, T. J. , McGinnity, P. , Coughlan, J. P. , Cross, T. F. , & Harrison, S. S. C. (2012). Morphological variability of Atlantic salmon *Salmo salar* and brown trout *Salmo trutta* in different river environments. Ecology of Freshwater Fish, 21(3), 420–432.

[ece33229-bib-0018] Dunn, P. O. , Armenta, J. K. , & Whittingham, L. A. (2015). Natural and sexual selection act on different axes of variation in avian plumage color. Science Advances, 1, e1400155. 26601146 10.1126/sciadv.1400155 PMC4643820

[ece33229-bib-0019] Emlen, S. T. (1976). Lek organization and mating strategies in the bullfrog. Behavioral Ecology and Sociobiology, 1, 283–313.

[ece33229-bib-0020] Emlen, S. T. , & Oring, L. W. (1977). Ecology, sexual selection, and the evolution of mating systems. Science, 197(4300), 215–223. 327542 10.1126/science.327542

[ece33229-bib-0021] Ferguson, M. M. , Danzmann, R. G. , & Hutchings, J. A. (1991). Incongruent estimates of population differentiation among brook charr, *Salvelinus fontinalis*, from Cape Race, Newfoundland, Canada, based upon allozyme and mitochondrial DNA variation. Journal of Fish Biology, 39(sA), 79–85.

[ece33229-bib-0022] Fischer, J. , & Lindenmayer, D. B. (2007). Landscape modification and habitat fragmentation: A synthesis. Global Ecology and Biogeography, 16, 265–280.

[ece33229-bib-0023] Fisher, R. A. (1930). The genetical theory of natural selection. Oxford: Oxford University Press.

[ece33229-bib-0024] Fleming, I. A. (1998). Pattern and variability in the breeding system of Atlantic salmon (*Salmo salar*), with comparisons to other salmonids. Canadian Journal of Fisheries and Aquatic Sciences, 55(1), 59–76.

[ece33229-bib-0025] Franssen, N. R. , Harris, J. , Clark, S. R. , Schaefer, J. F. , & Stewart, L. K. (2013). Shared and unique morphological responses of stream fishes to anthropogenic habitat alteration. Proceedings of the Royal Society B: Biological Sciences, 280, 20122715. 23235710 10.1098/rspb.2012.2715 PMC3574318

[ece33229-bib-0026] Fraser, D. J. , Debes, P. V. , Bernatchez, L. , & Hutchings, J. A. (2014). Population size, habitat fragmentation, and the nature of adaptive variation in a stream fish. Proceedings of the Royal Society B: Biological Sciences, 281(1790), 20140270. 10.1098/rspb.2014.0370 PMC4123695 25056619

[ece33229-bib-0027] Fraser, D. J. , Weir, L. K. , Bernatchez, L. , Hansen, M. M. , & Taylor, E. B. (2011). Extent and scale of local adaptation in salmonid fishes: Review and meta‐analysis. Heredity, 106(3), 404–420. 21224881 10.1038/hdy.2010.167 PMC3131967

[ece33229-bib-0029] Giery, S. T. , Layman, C. A. , & Langerhans, R. B. (2015). Anthropogenic ecosystem fragmentation drives shared and unique patterns of sexual signal divergence among three species of Bahamian mosquitofish. Evolutionary Applications, 8, 679–691. 26240605 10.1111/eva.12275 PMC4516420

[ece33229-bib-0030] Gilpin, M. E. , & Soulé, M. E. (1986). Minimum viable populations: Processes of species extinctions In Soulé M. E. (Ed.), Conservation biology: The science of scarcity and diversity, 1st ed. (pp. 19–34). Sunderland, MA, USA: Sinauer Associates.

[ece33229-bib-0031] Gouveia, L. , & Rema, P. (2005). Effect of microalgal biomass concentration and temperature on ornamental goldfish (*Carassius auratus*) skin pigmentation. Aquaculture Nutrition, 11(1), 19–23.

[ece33229-bib-0032] Hamon, T. R. , & Foote, C. J. (2005). Concurrent natural and sexual selection in wild male sockeye salmon, *Oncorhynchus nerka* . Evolution, 59(5), 1104–1118. 16136808

[ece33229-bib-0033] Hanski, I. , & Gilpin, M. (1991). Metapopulation dynamics: Brief history and conceptual domain. Biological Journal of the Linnean Society, 42, 3–16.

[ece33229-bib-0034] Haugen, T. O. , Aass, P. , Stenseth, N. C. , & Vøllestad, L. A. (2008). Changes in selection and evolutionary responses in migratory brown trout following the construction of a fish ladder. Evolutionary Applications, 1, 319–335. 25567634 10.1111/j.1752-4571.2008.00031.x PMC3352441

[ece33229-bib-0035] Heinen‐Kay, J. L. , Noel, H. G. , Layman, C. A. , & Langerhans, R. B. (2014). Human‐caused habitat fragmentation can drive rapid divergence of male genitalia. Evolutionary Applications, 7(10), 1252–1267. 25558285 10.1111/eva.12223 PMC4275096

[ece33229-bib-0036] Hendry, A. P. , Kinnison, M. T. , & Reznick, D. N. (2006). Parallel evolution of the sexes? Effects of predation and habitat features on the size and shape of wild guppies. Journal of Evolutionary Biology, 19, 741–754. 16674571 10.1111/j.1420-9101.2005.01061.x

[ece33229-bib-0037] Hutchings, J. A. (1993). Adaptive life histories effected by age‐specific survival and growth rate. Ecology, 74(3), 673–684.

[ece33229-bib-0038] Hutchings, J. A. (1996). Adaptive phenotypic plasticity in brook trout, *Salvelinus fontinalis*, life histories. Ecoscience, 3(1), 25–32.

[ece33229-bib-0039] Ishikawa, T. , & Gumiri, S. (2006). Dissolved organic carbon concentration of a natural water body and its relationship to water color in Central Kalimantan. Indonesia. Limnology, 7(2), 143–146.

[ece33229-bib-0040] Johnson, J. B. (2001). Adaptive life‐history evolution in the livebearing fish *Brachyrhaphis rhabdophora*: Genetic basis for parallel divergence in age and size at maturity and a test of predator‐induced plasticity. Evolution, 55(7), 1486–1491. 11525470 10.1111/j.0014-3820.2001.tb00668.x

[ece33229-bib-0041] Kelley, J. L. , Bree, P. , Cummins, G. H. , & Shand, J. (2012). Changes in the visual environment affect colour signal brightness and shoaling behaviour in a freshwater fish. Animal Behaviour, 83, 783–791.

[ece33229-bib-0042] Koskinen, M. T. , Haugen, T. O. , & Primmer, C. R. (2002). Contemporary fisherian life history evolution in small salmonid populations. Nature, 419, 826–830. 12397355 10.1038/nature01029

[ece33229-bib-0043] Langerhans, R. B. , Chapman, L. J. , & Dewitt, T. J. (2007). Complex phenotype ‐ environment associations revealed in an East African cyprinid. European Society for Evolutionary Biology, 20, 1171–1181. 10.1111/j.1420-9101.2007.01282.x 17465926

[ece33229-bib-0044] Langerhans, R. B. , & Dewitt, T. J. (2004). Shared and unique features of evolutionary diversification. The American Naturalist, 164(3), 335–349. 10.1086/422857 15478089

[ece33229-bib-0045] Lenth, R. V. (2016). Least‐squares means: The R package lsmeans. Journal of Statistical Software, 69(1), 1–33.

[ece33229-bib-0046] Letcher, B. H. , Nislow, K. H. , Coombs, J. A. , O'Donnell, M. J. , & Debreuil, T. L. (2007). Population response to habitat fragmentation in a stream‐dwelling brook trout population. PLoS ONE, 2(11), e1139. 18188404 10.1371/journal.pone.0001139 PMC2190617

[ece33229-bib-0047] Martinez, K. , & Cupitt, J. (2005). VIPS ‐ a highly tuned image processing software architecture (pp. 574–577). *Proceedings of the 2005 International Conference on Image Processing*, 2.

[ece33229-bib-0048] McCormick, J. H. , Hokanson, K. E. F. , & Jones, B. R. (1972). Effects of temperature on growth and survival of young brook trout, *Salvelinus fontinalis* . Journal of the Fisheries Research Board of Canada, 29(8), 1107–1112.

[ece33229-bib-0049] Murphy, S. M. , Battocletti, A. H. , Tinghitella, R. M. , Wimp, G. M. , & Ries, L. (2016). Complex community and evolutionary responses to habitat fragmentation and habitat edges: What can we learn from insect science? Current Opinion in Insect Science, 14, 61–65. 27436648 10.1016/j.cois.2016.01.007

[ece33229-bib-0050] Nitychoruk, J. M. , Gutowsky, L. F. G. , Harrison, P. M. , Hossie, T. J. , Power, M. , & Cooke, S. J. (2013). Sexual and seasonal dimorphism in adult adfluvial bull trout. Canadian Journal of Zoology, 91, 480–488.

[ece33229-bib-0051] Palkovacs, E. P. , Kinnison, M. T. , Correa, C. , Dalton, C. M. , & Hendry, A. P. (2012). Fates beyond traits: Ecological consequences of human‐induced trait change. Evolutionary Applications, 5, 183–191. 25568040 10.1111/j.1752-4571.2011.00212.x PMC3353338

[ece33229-bib-0052] Panhuis, T. M. , Butlin, R. , Zuk, M. , & Tregenza, T. (2001). Sexual selection and speciation. Trends in Ecology & Evolution, 16(7), 364–371. 11403869 10.1016/s0169-5347(01)02160-7

[ece33229-bib-0053] Pease, A. A. , Gonzalez‐Diaz, A. A. , Rodiles‐Hernandez, R. , & Winemiller, K. O. (2012). Functional diversity and trait – environment relationships of stream fish assemblages in a large tropical catchment. Freshwater Biology, 57, 1060–1075.

[ece33229-bib-0054] Pilastro, A. , Scaggiante, M. , & Rasotto, M. B. (2002). Individual adjustment of sperm expenditure accords with sperm competition theory. Proceedings of the National Academy of Sciences of the United States of America, 99(15), 9913–9915. 12107282 10.1073/pnas.152133499 PMC126598

[ece33229-bib-0055] Quinn, T. P. , Adkison, M. D. , & Ward, M. B. (1996). Behavioral tactics of male sockeye salmon (*Oncorhynchus nerka*) under varying operational sex ratios. Ethology, 102(4), 304–322.

[ece33229-bib-0056] Quinn, T. P. , & Foote, C. J. (1994). The effects of body size and sexual dimorphism on reproductive behaviour of sockeye salmon. Animal Behaviour, 48, 751–761.

[ece33229-bib-0057] Quinn, T. P. , Wetzel, L. , Bishop, S. , Overberg, K. , & Rogers, D. E. (2001). Influence of breeding habitat on bear predation and age at maturity and sexual dimorphism of sockeye salmon populations. Canadian Journal of Zoology, 79, 1782–1793.

[ece33229-bib-0058] R Core Team (2015). R: A language and environment for statistical computing, Vienna, Austria: R Foundation for Statistical Computing.

[ece33229-bib-0501] Ramstad, K. M. , Woody, C. A. , & Allendorf, F. W. (2010). Recent local adaptation of sockeye salmon to glacial spawning habitats. Evolutionary Ecology, 24(2), 391–411.

[ece33229-bib-0059] Reed, D. H. , O'Grady, J. J. , Brook, B. W. , Ballou, J. D. , & Frankham, R. (2003). Estimates of minimum viable population sizes for vertebrates and factors influencing those estimates. Biological Conservation, 113(1), 23–34.

[ece33229-bib-0060] Riddell, B. E. , & Leggett, W. C. (1981). Evidence of an adaptive basis for geographic variation in body morphology and time of downstream migration of juvenile Atlantic salmon (*Salmo salar*). Canadian Journal of Fisheries and Aquatic Science, 38, 308–320.

[ece33229-bib-0061] Rohlf, F. J. (2014). Morphometrics at SUNY Stony Brook. Retrieved from http://life.bio.sunysb.edu/morph/

[ece33229-bib-0062] Romano, A. , Costanzo, A. , Rubolini, D. , Saino, N. , & Møller, A. P. (2016). Geographical and seasonal variation in the intensity of sexual selection in the barn swallow *Hirundo rustica*: A meta‐analysis. Biological Reviews, https://doi.org/10.1111/brv.12297 10.1111/brv.12297 27615554

[ece33229-bib-0063] Schneider, C. A. , Rasband, W. S. , & Eliceiri, K. W. (2012). NIH Image to ImageJ: 25 years of image analysis. Nature Methods, 9(7), 671–675. 22930834 10.1038/nmeth.2089 PMC5554542

[ece33229-bib-0064] Seehausen, O. , Van Alphen, J. J. , & Witte, F. (1997). Cichlid fish diversity threatened by eutrophication that curbs sexual selection. Science, 277(5333), 1808–1811.

[ece33229-bib-0065] Taylor, E. B. (1991). A review of local adaptation in Salmonidae, with particular reference to Pacific and Atlantic salmon. Aquaculture, 98(1), 185–207.

[ece33229-bib-0066] Valentin, A. E. , Penin, X. , Chanut, J. P. , Sévigny, J. M. , & Rohlf, F. J. (2008). Arching effect on fish body shape in geometric morphometric studies. Journal of Fish Biology, 73(3), 623–638.

[ece33229-bib-0067] Weatherly, A. H. , & Gill, H. S. (1987). The biology of fish growth. London: Academic Press.

[ece33229-bib-0068] Wedekind, C. , Jacob, A. , Evanno, G. , Nusslé, S. , & Müller, R. (2008). Viability of brown trout embryos positively linked to melanin‐based but negatively to carotenoid‐based colours of their fathers. Proceedings of the Royal Society B: Biological Sciences, 275, 1737–1744. 18445560 10.1098/rspb.2008.0072 PMC2453293

[ece33229-bib-0069] Weir, L. K. , Grant, J. W. A. , & Hutchings, J. A. (2011). The influence of operational sex ratio on the intensity of competition for mates. The American Naturalist, 177(2), 167–176. 10.1086/657918 21460553

[ece33229-bib-0070] Weir, L. K. , Kindsvater, H. K. , Young, K. A. , & Reynolds, J. D. (2016). Sneaker males affect fighter male body size and sexual size dimorphism in salmon. The American Naturalist, 188(2), 264–271. 10.1086/687253 27420790

[ece33229-bib-0071] Wellborn, G. A. , & Langerhans, R. B. (2015). Ecological opportunity and the adaptive diversification of lineages. Ecology and Evolution, 5(1), 176–195. 25628875 10.1002/ece3.1347 PMC4298445

[ece33229-bib-0072] Wells, Z. R. R. , McDonnell, L. H. , Chapman, L. J. , & Fraser, D. J. (2016). Limited variability in upper thermal tolerance among pure and hybrid populations of a cold‐water fish. Conservation Physiology, 4, cow063 https://doi.org/10.1093/conphys/cow063 27990291 10.1093/conphys/cow063 PMC5156897

[ece33229-bib-0073] Westley, P. A. , Conway, C. M. , & Fleming, I. A. (2012). Phenotypic divergence of exotic fish populations is shaped by spatial proximity and habitat differences across an invaded landscape. Evolutionary Ecology Research, 14(2), 147–167.

[ece33229-bib-0074] White, H. (1980). A heteroskedasticity‐consistent covariance matrix estimator and a direct test for heteroskedasticity. Econometrica, 48(4), 817–838.

[ece33229-bib-0075] Willi, Y. , Buskirk, B. , Schmid, B. , & Fischer, M. (2007). Genetic isolation of fragmented populations is exacerbated by drift and selection. Journal of Evolutionary Biology, 20, 534–542. 17305819 10.1111/j.1420-9101.2006.01263.x

[ece33229-bib-0076] Willi, Y. , & Hoffmann, A. A. (2012). Microgeographic adaptation linked to forest fragmentation and habitat quality in the tropical fruit fly *Drosophila birchii* . Oikos, 121, 1627–1637.

[ece33229-bib-0077] Wood, J. L. A. , Belmar‐Lucero, S. , & Hutchings, J. A. (2014). Relationship of habitat variability to population size in a stream fish. Ecological Applications, 24(5), 1085–1100. 25154098 10.1890/13-1647.1

[ece33229-bib-0078] Wood, J. L. A. , & Fraser, D. J. (2015). Similar plastic responses to elevated temperature among different‐sized brook trout populations. Ecology, 96(4), 1010–1019. 26230021 10.1890/14-1378.1

[ece33229-bib-0079] Wood, J. L. A. , Tezel, D. , Joyal, D. , & Fraser, D. J. (2015). Population size is weakly related to quantitative genetic variation and trait differentiation in a stream fish. Evolution, 69(9), 2303–2318. 26207947 10.1111/evo.12733

[ece33229-bib-0080] Wood, J. L. A. , Yates, M. C. , & Fraser, D. J. (2016). Are heritability and selection related to population size in nature? Meta‐analysis and conservation implications Evolutionary Applications, 9(5), 640–657. 27247616 10.1111/eva.12375 PMC4869407

[ece33229-bib-0081] WWF (2016). Living planet report 2016: Risk and resilience in a new era. WWF International, Gland, Switzerland.

[ece33229-bib-0082] Young, K. A. (2005). Life‐history variation and allometry for sexual size dimorphism in Pacific salmon and trout. Proceedings of the Royal Society B: Biological Sciences, 272(1559), 167–172. 15695207 10.1098/rspb.2004.2931 PMC1634950

[ece33229-bib-0083] Ytrestøyl, T. , Struksnæs, G. , Koppe, W. , & Bjerkeng, B. (2005). Effects of temperature and feed intake on astaxanthin digestibility and metabolism in Atlantic salmon, *Salmo salar* . Comparative Biochemistry and Physiology Part B: Biochemistry and Molecular Biology, 142(4), 445–455. 10.1016/j.cbpb.2005.09.004 16242366

[ece33229-bib-0084] Zelditch, M. L. , Swiderski, D. L. , Sheets, H. D. , & Fink, W. L. (2004). Geometric morphometrics for biologists: A primer. San Diego, California, US: Elsevier Academic Press.

