# Supplementary material for: The evolutionary consequences of habitat fragmentation: Body morphology and coloration differentiation among brook trout populations of varying size
Source: Ecol Evol. 2017 Jul 27;7(17):6850–62. doi: 10.1002/ece3.3229 (PMC5587476; doi:10.1002/ece3.3229)
Supplement: Supplementary file 1 [file ECE3-7-6850-s001.docx]

**APPENDIX S1**

**Additional Tables and Figures Referred to In-text**

**Table S1.** Overall trait means across 14 brook trout populations in Cape Race, Newfoundland, Canada.

| Population | Mass (g) | Length (mm) | Condition Factor | Pectoral Fin/Body Length (%) | Pelvic Fin/Body Length (%) |
| --- | --- | --- | --- | --- | --- |
| BC | 16.32 | 110.87 | 1.14 | 17.28 | 13.02 |
| CC | 39.51 | 149.38 | 1.09 | 16.71 | 12.72 |
| DY | 26.12 | 129.38 | 1.18 | 17.19 | 12.93 |
| FW | 14.68 | 104.74 | 1.19 | 16.64 | 12.43 |
| HM | 35.41 | 137.64 | 1.31 | 16.66 | 12.95 |
| LBF | 32.28 | 135.90 | 1.21 | 17.69 | 13.51 |
| LC | 17.14 | 109.44 | 1.27 | 15.71 | 12.17 |
| LO | 13.93 | 105.94 | 1.13 | 16.68 | 12.64 |
| PD | 13.35 | 101.13 | 1.29 | 14.87 | 12.56 |
| STBC | 16.00 | 109.78 | 1.15 | 15.28 | 11.82 |
| UC | 16.90 | 110.22 | 1.25 | 15.40 | 12.19 |
| UO | 24.56 | 127.16 | 1.16 | 16.95 | 13.23 |
| WC | 33.60 | 141.05 | 1.70 | 17.92 | 13.78 |
| WN | 27.46 | 130.63 | 1.16 | 16.79 | 13.03 |

**Table S1 (cont.)**

| Population | RW1  (body depth) | RW2 (dorsal hump) | RW3 (caudal peduncle) | RW4 (head  size) | Red Area/  Body Area (%) | Red Saturation | Spot Number |
| --- | --- | --- | --- | --- | --- | --- | --- |
| BC | -4.98E-04 | -3.05E-03 | 1.09E-02 | 8.61E-03 | 7.33 | 106.73 | 12 |
| CC | -1.16E-02 | 1.34E-03 | 1.26E-02 | -2.67E-03 | 12.95 | 112.02 | 21 |
| DY | 2.10E-02 | -2.65E-04 | 6.77E-03 | 6.96E-03 | 18.76 | 141.49 | 16 |
| FW | -3.52E-03 | 5.24E-04 | -3.82E-03 | 5.99E-03 | 12.74 | 134.45 | 10 |
| HM | 1.32E-02 | -5.55E-03 | -5.12E-03 | 6.87E-03 | 15.80 | 119.26 | 9 |
| LBF | 6.98E-03 | -9.49E-03 | 9.51E-04 | -5.52E-03 | 12.61 | 139.16 | 15 |
| LC | 6.54E-03 | -1.70E-02 | -2.80E-03 | 1.97E-03 | 10.07 | 123.12 | 7 |
| LO | 1.51E-03 | 1.13E-02 | -8.64E-03 | 4.76E-03 | 18.83 | 133.72 | 9 |
| PD | -3.52E-03 | 5.46E-03 | -8.43E-03 | 1.98E-03 | 17.21 | 132.17 | 8 |
| STBC | 4.73E-05 | -8.57E-03 | -6.47E-03 | 4.17E-03 | 8.33 | 114.38 | 9 |
| UC | -3.27E-03 | -1.13E-02 | -2.12E-03 | 9.69E-03 | 17.08 | 119.65 | 6 |
| UO | -8.91E-04 | 5.84E-03 | -3.30E-03 | -1.66E-03 | 18.35 | 138.70 | 12 |
| WC | -2.56E-03 | 7.27E-03 | -2.87E-03 | 2.12E-03 | 9.52 | 132.01 | 9 |
| WN | 1.84E-03 | 1.89E-02 | 6.04E-04 | -8.06E-03 | 14.25 | 139.68 | 16 |

**Table S2.** Overall trait means for females and males in 14 brook trout populations in Cape Race, Newfoundland, Canada.

| Trait Category | Trait | Female Mean | Male Mean |
| --- | --- | --- | --- |
| Body size | Mass (g) | 24.96 | 24.58 |
| Body size | Length (mm) | 125.00 | 123.64 |
| Body size | Condition factor | 1.18 | 1.18 |
| Body shape | RW1 (body depth) | -1.05E-02 | 1.01E-02 |
| Body shape | RW2 (dorsal hump) | -2.64E-03 | 2.51E-03 |
| Body shape | RW3 (caudal peduncle) | -2.28E-03 | 2.19E-03 |
| Body shape | RW4 (head size) | 6.68E-04 | -6.44E-04 |
| Colouration | Red Area/Body Area (%) | 12.64 | 13.57 |
| Colouration | Red Saturation | 118.89 | 138.30 |
| Colouration | Spot number | 12.68 | 13.02 |
| Fin Length | Pectoral Fin/Body Length (%) | 20.34 | 21.50 |
| Fin Length | Pelvic Fin/Body Length (%) | 16.00 | 16.33 |

**Table S3.**  Environmental and demographic variables in 14 brook trout populations in Cape Race, Newfoundland, Canada, collected from 2012-2015 (Bernos & Fraser 2016).

| Population | pH | Depth (cm) | Temperature (°C) | Velocity (m/s) | Mean N_c_ | Mean N_b_ | OSR (M/F) |
| --- | --- | --- | --- | --- | --- | --- | --- |
| BC | 6.29 | 15.31 | 14.17 | 0.29 | 4693 (4404-6132) | 355 (267-567) | 3.34 |
| CC | 6.35 | 17.82 | 14.66 | 0.31 | 1862 (1471-5246) | 74 (65-99) | 9.75 |
| DY | 5.88 | 25.02 | 14.39 | 0.04 | 116 (84-179) | 10 (3-34) | 3.43 |
| FW | 6.57 | 18.45 | 14.88 | 0.38 | 5367 | 219 | 5.80 |
| HM | 6.17 | 46.40 | 12.25 | 0 | 66 (52-80) | 5 (3-5) | 1.15 |
| LBF | 6.06 | 13.26 | 11.61 | 0.14 | 1184 (877-1383) | 52 (34-83) | 9.20 |
| LC | 6.15 | 9.60 | 11.15 | 0.19 | 338 (250-798) | 31 (11-117) | 1.17 |
| LO | 6.65 | 21.24 | 16.34 | 0.48 | 470 (372-625) | 44 (23-188) | 9.33 |
| PD | 5.30 | 18.91 | 14.01 | 0.27 | 992 | 79 | 4.85 |
| STBC | 6.07 | 21.34 | 9.40 | 0.04 | 917 (587-1405) | 28 (14-54) | 6.50 |
| UC | 5.17 | 24.62 | 11.03 | 0.04 | 65 (49-79) | 20 (13-48) | 1.50 |
| UO | 6.41 | 22.16 | 15.88 | 0.17 | 2569 (1949-3835) | 62 (41-87) | 9.90 |
| WC | 6.02 | 17.01 | 12.84 | 0.13 | 783 (530-1148) | 31 (21-52) | 3.73 |
| WN | 6.69 | 22.68 | 13.98 | 0.43 | 7801 (6713-10032) | 178 (110-267) | 7.96 |

**Table S4.** *F*-values (p < 0.05) from linear mixed models with traits as dependent variables and with habitat characteristics, sex, census population size (**N_b_**), and OSR as predictor variables for 14 brook trout populations in Cape Race, Newfoundland, Canada. Condition factor and spot number have overall results only as sex was not significant. Models were done for both sexes combined (indicated with “O”) as well as separated (indicated with “F” and “M”). “+” or “–” in front of values represents a positive or negative relationship (for sex, relationship is displayed in reference to males). NS represents insignificant results, and N/A means not applicable.

| Trait | pH:Temp | Temp:Velocity | pH | Depth | Velocity | Temp | Sex | Nb | OSR |
| --- | --- | --- | --- | --- | --- | --- | --- | --- | --- |
| O: | NS | 1.7504 | NS | NS | (-) 3.0080 | (+) 0.0080 | (-) 9.5564 | NS | NS |
| Mass F: | NS | 2.0334 | NS | NS | (-) 2.2839 | (+) 0.3075 | N/A | NS | (+) 3.1184 |
| M: | NS | 2.5496 | NS | NS | (-) 3.7648 | (+) 0.0107 | N/A | NS | NS |
| O: | NS | 1.2618 | NS | NS | (-) 1.9429 | (+) 0.0237 | (-) 10.8169 | NS | NS |
| Length F: | NS | 1.8924 | NS | NS | (-) 1.4710 | (+) 0.0679 | N/A | NS | (+) 3.8015 |
| M: | NS | 1.6757 | NS | NS | (-) 2.4362 | (+) 0.1321 | N/A | NS | NS |
| Condition O: | NS | NS | (-) 13.25 | NS | NS | NS | NS | NS | NS |
| O: | NS | NS | NS | NS | NS | NS | (+) 986.9029 | NS | (-) 6.7731 |
| RW1 F: | NS | NS | NS | NS | NS | NS | N/A | NS | NS |
| M: | NS | NS | NS | NS | NS | NS | N/A | NS | (-) 9.7182 |
| O: | NS | NS | NS | NS | NS | (+) 22.710 | (+) 63.6710 | NS | NS |
| RW2 F: | NS | NS | NS | NS | NS | (+) 26.537 | N/A | NS | NS |
| M: | NS | NS | NS | NS | NS | (+) 14.4732 | N/A | NS | NS |
| O: | NS | NS | NS | NS | NS | NS | (+) 62.5886 | NS | NS |
| RW3 F: | NS | NS | NS | NS | NS | NS | N/A | NS | NS |
| M: | NS | NS | NS | NS | NS | NS | N/A | NS | NS |
| O: | NS | NS | NS | (-) 2.4601 | NS | NS | (-) 5.4647 | (+) 0.6477 | (+) 12.2757 |
| RW4 F: | NS | NS | NS | NS | NS | NS | N/A | (+) 0.0051 | (+)8.4886 |
| M: | NS | NS | NS | (-) 0.5429 | NS | NS | N/A | (+) 0.1555 | (+) 8.3216 |
| O: | NS | NS | NS | (+) 7.1510 | NS | (+) 9.6239 | (+) 4.6355 | (-) 27.2992 | NS |
| Red Area F: | 0.3376 | 4.2464 | (-) 5.8002 | (+) 7.6573 | (+) 0.0978 | (+) 34.7793 | N/A | (-) 19.1734 | NS |
| M: | NS | NS | (-) 3.1497 | NS | NS | (+) 8.4288 | N/A | (-) 18.9450 | (+) 0.0968 |
| Red O: | NS | NS | NS | NS | NS | NS | (+) 362.0075 | NS | NS |
| Saturation F: | NS | NS | NS | NS | NS | NS | N/A | NS | NS |
| M: | NS | NS | NS | NS | NS | NS | N/A | (-) 5.7490 | NS |
| Spots O: | NS | NS | NS | NS | NS | NS | N/A | NS | (+) 8.3916 |
| Pectoral O: | NS | NS | NS | NS | NS | NS | (+) 58.2210 | NS | NS |
| Fin F: | NS | 0.8323 | NS | NS | (+) 6.6123 | (+) 0.8745 | N/A | NS | NS |
| M: | NS | NS | NS | NS | NS | NS | N/A | NS | NS |
| Pelvic O: | NS | 3.0472 | NS | NS | (+) 20.2074 | (-) 3.9688 | (+) 0.8483 | NS | NS |
| Fin F: | NS | 10.3228 | NS | (+) 16.0433 | (+) 23.5097 | (+) 0.2002 | N/A | NS | (+) 85.5298 |
| M: | NS | 5.649 | NS | NS | (+) 2.1359 | (-) 7.2914 | N/A | NS | NS |

**Table S5.** *F*-values (p < 0.05) from linear mixed models of best fit for each phenotypic trait, with habitat characteristics, sex, census population size (**N_c_**), and OSR as predictor variables for 14 brook trout populations in Cape Race, Newfoundland, Canada. Condition factor and spot number have overall results only as sex was not significant. Models were done for both sexes combined (indicated with “O”) as well as separated (indicated with “F” and “M”). “+” or “-” in front of values represents a positive or negative relationship (for sex, relationship is displayed in reference to males). NS represents insignificant results, and N/A means not applicable.

| Trait | pH:Temp | Temp:Velocity | pH | Depth | Velocity | Temperature | Sex | Nc | OSR |
| --- | --- | --- | --- | --- | --- | --- | --- | --- | --- |
| O: | NS | 8.7682 | NS | (+) 2.6631 | (-) 2.6631 | (+) 0.0125 | (-) 9.3712 | NS | (+) 3.5964 |
| Mass F: | NS | 7.7647 | NS | (+) 3.1333 | (-) 1.1601 | (+) 0.4472 | N/A | NS | (+) 5.5375 |
| M: | NS | 8.3551 | NS | (+) 1.4409 | (-) 4.0397 | (+) 0.0124 | N/A | NS | (+) 2.4573 |
| O: | NS | 6.7486 | NS | (+) 1.4786 | (-) 1.8530 | (+) 0.0396 | (-) 10.5990 | NS | (+) 4.5515 |
| Length F: | NS | 6.0924 | NS | (+) 1.7494 | (-) 0.7131 | (+) 0.0910 | N/A | NS | (+) 5.8272 |
| M: | NS | 3.7325 | NS | NS | (-) 3.2263 | (+) 0.1715 | N/A | NS | (+) 3.2598 |
| Condition O: | NS | NS | (-) 13.2500 | NS | NS | NS | NS | NS | NS |
| O: | NS | NS | NS | NS | NS | NS | (+) 986.9029 | NS | (-) 6.7731 |
| RW1 F: | NS | NS | NS | NS | NS | NS | N/A | NS | NS |
| M: | NS | NS | NS | NS | NS | NS | N/A | NS | (-) 9.7182 |
| O: | NS | NS | NS | NS | NS | (+) 22.7100 | (+) 63.6710 | NS | NS |
| RW2 F: | NS | NS | NS | NS | NS | (+) 26.5370 | N/A | NS | NS |
| M: | NS | NS | NS | NS | NS | (+) 14.4732 | N/A | NS | NS |
| O: | NS | NS | NS | NS | NS | NS | (+) 62.5886 | NS | NS |
| RW3 F: | NS | NS | NS | NS | NS | NS | N/A | NS | NS |
| M: | NS | NS | NS | NS | NS | NS | N/A | NS | NS |
| O: | NS | NS | NS | (-) 2.4336 | NS | NS | (-) 5.4600 | NS | (-) 12.1621 |
| RW4 F: | NS | NS | NS | NS | NS | NS | N/A | NS | (-) 8.4797 |
| M: | NS | NS | NS | (-) 0.5429 | NS | NS | N/A | (+) 0.1555 | (-) 8.3216 |
| O: | 0.2120 | 3.8903 | (-) 2.2715 | (+) 5.9265 | (+) 0.1005 | (+) 11.4452 | (+) 4.8657 | (-) 4.1947 | (+) 1.5809 |
| Red Area F: | 1.9321 | 4.2067 | (-) 4.5885 | (+) 9.2137 | (+) 0.0337 | (+) 45.2201 | N/A | (-) 21.3188 | NS |
| M: | 0.3187 | NS | (-) 2.9769 | (+) 3.5581 | NS | (-) 3.6279 | N/A | (-) 1.7603 | (+) 1.4698 |
| Red O: | NS | NS | NS | NS | NS | NS | (+) 362.0075 | NS | NS |
| Saturation F: | NS | NS | NS | NS | NS | NS | N/A | NS | NS |
| M: | NS | NS | NS | NS | NS | NS | N/A | NS | NS |
| Spots O: | NS | NS | NS | NS | NS | NS | NS | NS | NS |
| Pectoral O: | NS | NS | NS | NS | NS | NS | (+) 58.2210 | NS | NS |
| Fin F: | NS | 0.8323 | NS | NS | (+) 6.6123 | (+) 0.8745 | N/A | NS | NS |
| M: | NS | NS | NS | NS | NS | NS | N/A | NS | NS |
| Pelvic O: | NS | 5.6512 | NS | (+) 11.8457 | (+) 12.9409 | (-) 4.3748 | (+) 0.9906 | NS | NS |
| Fin F: | NS | 10.3228 | NS | (+) 16.0433 | (+) 23.5097 | (-) 0.2002 | N/A | NS | (+) 85.5298 |
| M: | NS | 5.6249 | NS | NS | (+) 2.1359 | (-) 7.2914 | N/A | NS | NS |

**Figure S1.** Cape Race, Newfoundland, Canada streams. From west to east: 1) Perdition (PD), 2) Freshwater (FW), 3) Lower Coquita (LC), 4) Upper Coquita (UC), 5) Hermitage (HM), 6) Bob’s Cove (BC), 7) Still There By Chance (STBC), 8) Whale Cove (WC), 9) Ditchy (DY), 10) Upper O’Beck (UO), 11) Lower O’Beck (LO), 12) Watern (WN), 13) Lower Blackfly (LBF), 14) Cripple Cove (CC).

**
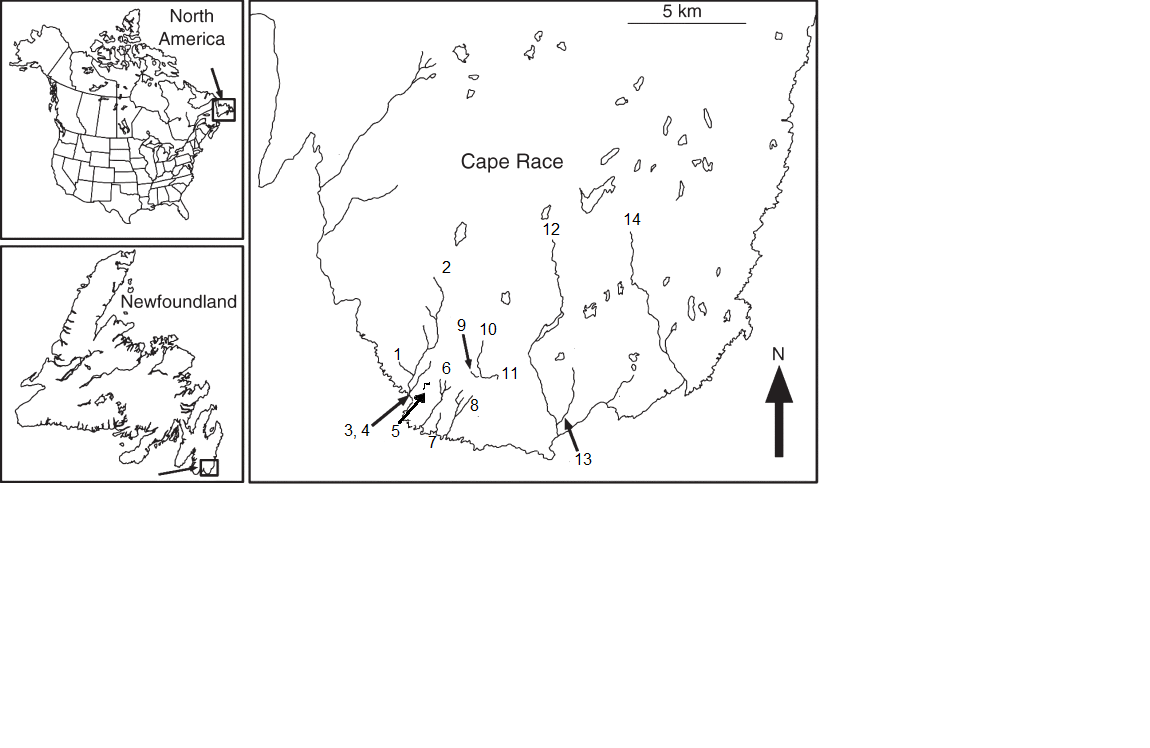
**

**Figure S2.** Landmarks for geometric morphometric analysis on brook trout.


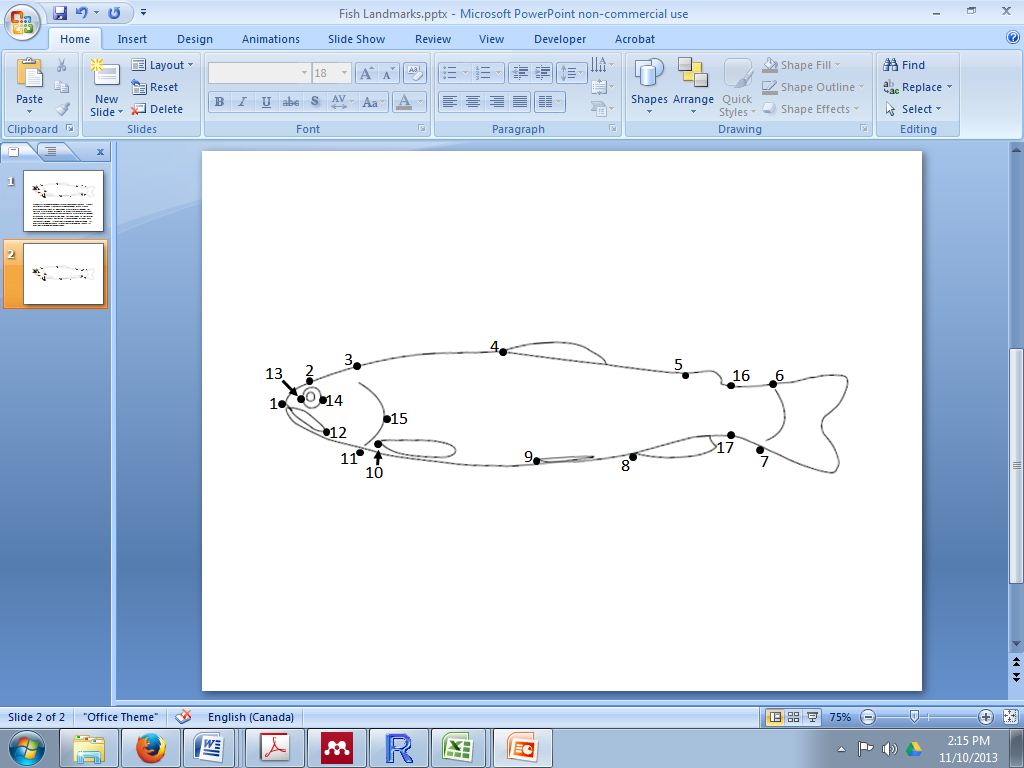


1, the most anterior part of body; 2, the head directly above midpoint of the eye; 3, the head directly above dorsal limit of operculum; 4, the anterior insertion point for dorsal fin; 5, the anterior limit of adipose fin; 6, the dorsal terminus of the caudal peduncle; 7, the ventral terminus of the caudal peduncle; 8, the anterior insertion point of the anal fin; 9, the anterior insertion point for the left pelvic fin; 10, the anterior insertion point for the left pectoral fin; 11, the meeting point of the gill plate and the ventral midline; 12, the most posterior point on upper mandible; 13, the most anterior point on the eye; 14, the most posterior point on the eye; 15, the most posterior point on the operculum; 16, the dorsal position above the thinnest part of the caudal peduncle; 17, the ventral position below the thinnest part of the caudal peduncle.

**Figure S3.** Female and male means of (left to right) mass, RW4 (head size), length, pectoral fin length/total body length, RW3, and pelvic fin length/total body length across 14 brook trout populations in Cape Race, Newfoundland, Canada, increasing in population size (N_b_) along the x-axes. Trait means depicted with 95% confidence intervals.


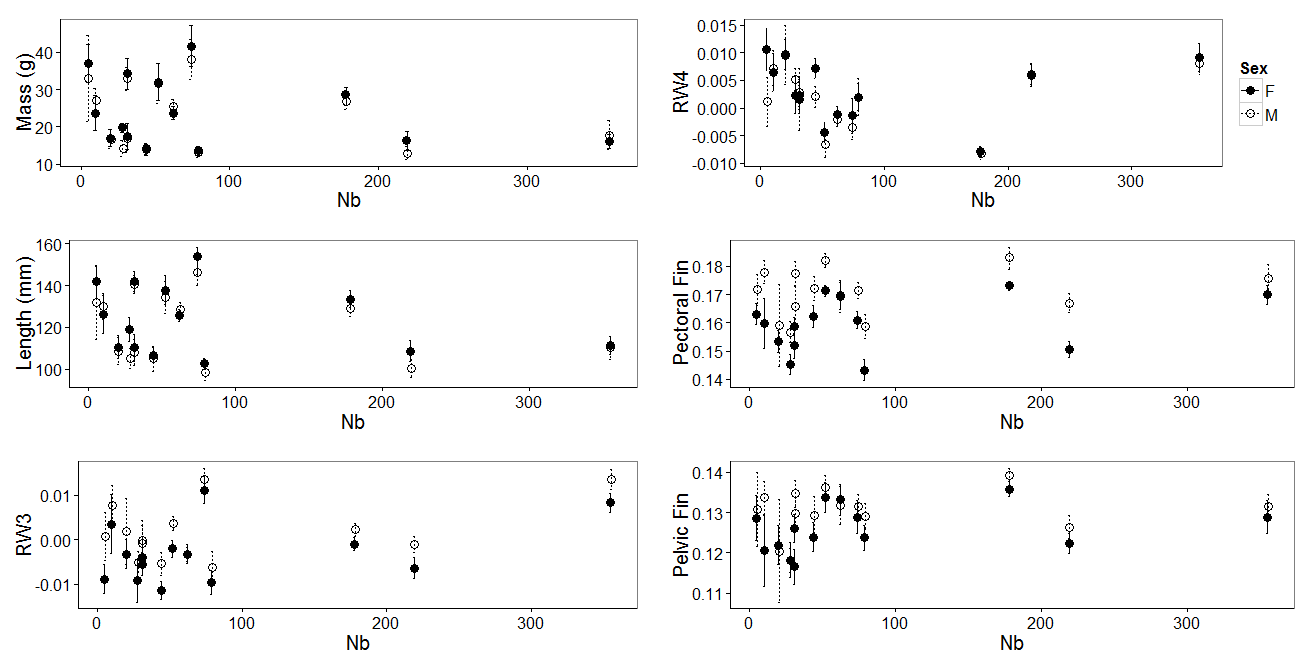


**Figure S4.** Examples of mean trait and habitat interactions 14 brook trout populations in Cape Race, Newfoundland, Canada. From left to right: mass across stream velocities, length across stream velocities, condition factor across stream pH (CF), RW4 across stream OSRs, red area/total body area across stream depths, red saturation across stream velocities, pectoral fin length/total body length across stream temperatures, pelvic fin length/total body length across stream velocities, and pelvic fin length/total body length across stream OSRs. Trait means depicted with 95% confidence intervals.
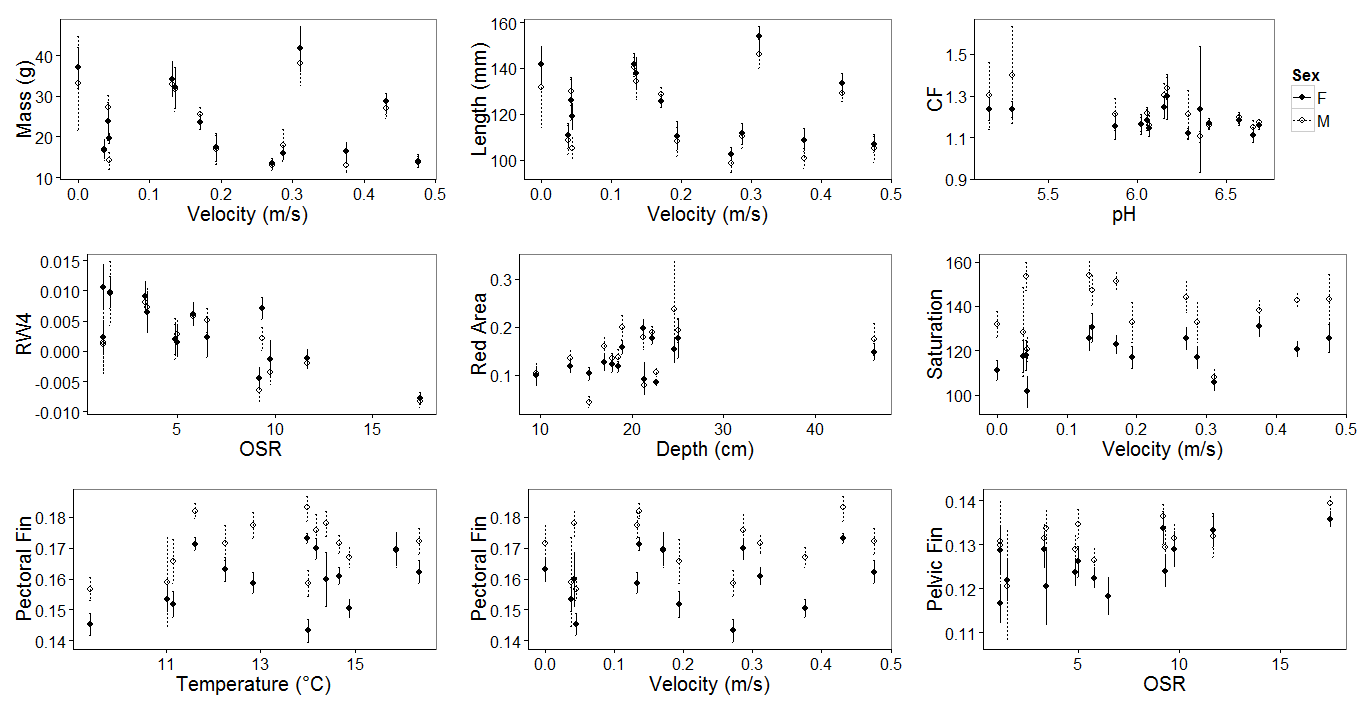


**APPENDIX S2**

**White’s Tests for Heteroscedasticity**

**Table S6**. White’s Test with N_b_ and phenotypic trait means across populations.

| Traits | Female | Female P value | Male | Male P value |
| --- | --- | --- | --- | --- |
| Mass Mean | 1.71 | 0.42 | 2.52 | 0.28 |
| Length Mean | 1.99 | 0.37 | 2.82 | 0.24 |
| Condition Factor Mean | 4.15 | 0.13 | 1.18 | 0.55 |
| RW1 Mean | 1.07 | 0.59 | 1.42 | 0.49 |
| RW2 Mean | 1.50 | 0.47 | 1.70 | 0.43 |
| RW3 Mean | 0.40 | 0.82 | 0.65 | 0.72 |
| RW4 Mean | 2.10 | 0.35 | 2.41 | 0.30 |
| Red Area Mean | 1.23 | 0.54 | 1.22 | 0.54 |
| Saturation Mean | 0.17 | 0.92 | 1.00 | 0.61 |
| Spot Number Mean | 0.77 | 0.68 | 0.79 | 0.67 |
| Pectoral Fin Mean | 4.72 | 0.09 | 1.38 | 0.50 |
| Pelvic Fin Mean | 2.18 | 0.34 | 0.33 | 0.85 |

**Table S7**. White’s Test with N_b_ and phenotypic trait CVs across populations.

| Traits | Female | Female *P-*value | Male | Male *P-*value |
| --- | --- | --- | --- | --- |
| Mass CV | 4.85 | 0.09 | 1.86 | 0.40 |
| Length CV | 8.85 | 0.01* | 2.50 | 0.29 |
| Condition Factor CV | 3.99 | 0.14 | 0.66 | 0.72 |
| RW1 CV | 0.56 | 0.76 | 2.10 | 0.35 |
| RW2 CV | 1.29 | 0.53 | 0.52 | 0.77 |
| RW3 CV | 3.15 | 0.21 | 2.12 | 0.35 |
| RW4 CV | 1.12 | 0.57 | 1.27 | 0.53 |
| Red Area CV | 0.46 | 0.79 | 0.32 | 0.85 |
| Saturation CV | 0.23 | 0.89 | 1.64 | 0.44 |
| Spot Number CV | 0.39 | 0.82 | 1.96 | 0.38 |
| Pectoral Fin CV | 0.63 | 0.73 | 0.48 | 0.79 |
| Pelvic Fin CV | 0.38 | 0.83 | 0.63 | 0.73 |

**APPENDIX S3**

**Variation Tests using Linear Mixed Models**

**Table S8.** *F*-values (p<0.05) for each phenotypic trait mean using habitat CVs as predictor variables. Symbol “-“ used for negative relationships.

| Traits | pH CV | Depth CV | Velocity CV | Temp CV | Sex | N_b_ |
| --- | --- | --- | --- | --- | --- | --- |
| Mass mean |  |  |  |  |  |  |
| Length mean | 4.6077 | (-) 10.6450 |  | (-) 5.7088 | (-) 7.5149 |  |
| Condition mean |  |  |  |  |  |  |
| RW1 mean |  |  |  |  | 220.22 |  |
| RW2 mean | 7.4285 |  |  |  | 43.4488 |  |
| RW3 mean |  |  |  |  | 74.371 |  |
| RW4 mean | 2.9193 |  | 2.9697 | 2.2399 |  |  |
| Red area mean |  |  | 8.1434 | 10.6119 |  |  |
| Saturation mean | 3.805 |  |  |  | 68.866 |  |
| Spot number mean |  |  |  |  | 66.809 |  |
| Pectoral fin mean |  |  |  |  | 12.9 |  |
| Pelvic fin mean |  |  |  |  |  |  |

**Table S9.** *F*-values (p<0.05) for each phenotypic trait CV using habitat CVs as predictor variables. Symbol “-“ used for negative relationships.

| Traits | pH CV | Depth CV | Velocity CV | Temp CV | Sex | N_b_ |
| --- | --- | --- | --- | --- | --- | --- |
| Mass CV |  |  |  |  |  | 7.6886 |
| Length CV |  |  |  |  | 5.1713 | 5.8261 |
| Condition CV |  |  |  |  |  |  |
| RW1 CV |  |  |  |  |  |  |
| RW2 CV | 10.4175 |  | (-) 1.5805 |  | 6.1401 |  |
| RW3 CV |  |  |  |  |  |  |
| RW4 CV |  |  |  |  |  |  |
| Red area CV |  |  |  |  |  |  |
| Saturation CV |  |  |  |  |  |  |
| Spot number CV |  | 11.7107 | (-) 5.3947 | (-) 0.2922 |  | (-) 3.3884 |
| Pectoral fin CV |  |  |  |  |  |  |
| Pelvic fin CV |  |  |  |  |  |  |

As a consistent trend was not seen between habitat variation (represented by coefficient of variation; CV) and phenotypic trait means and CVs, the impact of habitat variation on results was not considered in the main body of text.

**APPENDIX S4**

**Determination of a genetic component to phenotypic trait differentiation among Cape Race brook trout populations**

To determine if the phenotypic trait differentiation observed among Cape Race trout populations has a genetic component, we examined the correlation between the female and male body size (mass and length) in the wild versus when populations were reared under common environmental conditions in captivity over their lifetime (data are from a concurrent study, DJ Fraser et al., unpublished). A large number of full sibling families from 9 of 14 wild populations represented in this study were generated simultaneously in captivity from eggs and sperm collected and crossed from wild adults in 2011 and reared to maturation in 2014. The captive populations experienced the same densities, feed regimes, tank space, temperature, pH and dissolved oxygen throughout the experiment (see details of early rearing and cross design in Wood & Fraser 2015; Wood et al. 2015). The majority of trout (>95%) from different captive populations matured at the same age in the fall of 2014. Female mass, female length, male mass, and male length were all found to be positively correlated between captive and wild populations, although size in captivity was much larger than in the wild.

**Figure S5.** Female mass, female length, male mass, and male length of wild and captive populations with the adjusted R^2^.


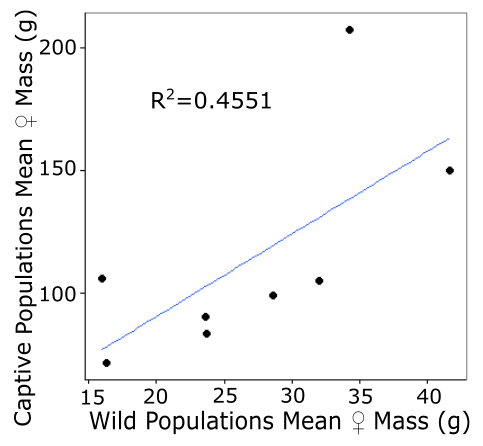

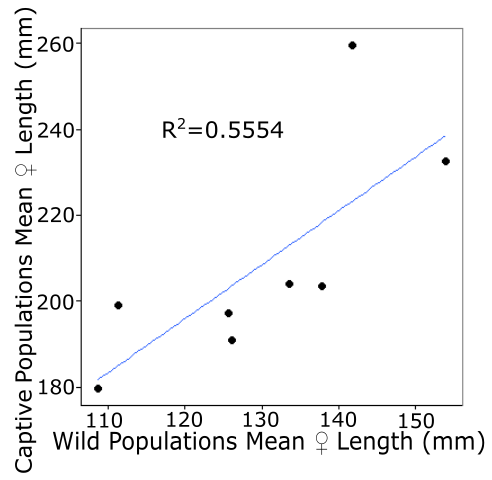


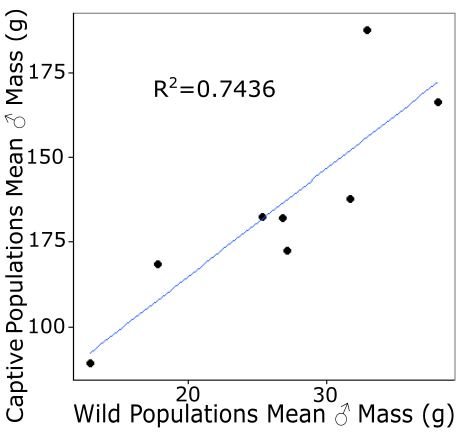

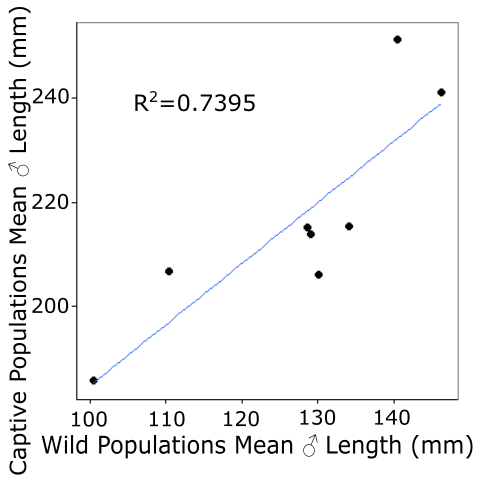


**APPENDIX S5**

**Models Used**

**Table S10.** Full models used to test inter-population trait variation and phenotype-environment associations through backwards step-wise model selection. Log likelihood comparisons were used to get the models of best fit.

| Test type | Full model |
| --- | --- |
| Inter-population trait variation | lm(Trait ~ Population + Sex + Population:Sex + Size) |
| Phenotype-environment associations | lmer(Trait ~ Size.correlate + Depth + Temperature + Velocity + pH + pH:Temperature + Temperature:Velocity + Sex + Nb + OSR+ (1\|Population)) |
